# *Basic pathway* decomposition of biochemical reaction networks within growing cells

**DOI:** 10.1016/j.isci.2023.108506

**Published:** 2023-11-22

**Authors:** Jay R. Walton, Paul A. Lindahl

**Affiliations:** 1 Department of Mathematics, Texas A&M University, College Station, TX 77843-3368, USA; 2 Department of Chemistry, Texas A&M University, College Station, TX 77843-3255, USA; 3 Department of Biochemistry and Biophysics, Texas A&M University, College Station, TX 77843, USA

**Keywords:** Biological sciences, Metabolic engineering, Network

## Abstract

This contribution treats linear, steady-state dynamics for a metabolic network within a growing cell. Admissible steady-state reaction fluxes are assumed to form a pointed, convex, polyhedral, conical subset of the stoichiometric null-space. A solution of the problem is defined to consist of a linear basis for the stoichiometric null-space consisting of admissible fluxes called *basic pathways*. The algorithm used to construct the set of basic pathways scales as a polynomial of the system size in contrast to the NP-hard algorithms employed in the traditional notions of solution named *extreme pathways*, *elementary flux modes*, *MEMos*, and *MinSpan*, and that therefore suffer from the curse of dimensionality. The basic pathways approach is applied to a metabolic network consisting of a simplified version of the TCA cycle coupled to glycolysis highlighting that each basic pathway has a readily understood chemical interpretation. Generic admissible pathways are simply expressed in terms of basic pathways.

## Highlights

• Steady-state analysis of metabolic network in growing cell
• Defined *basic pathways* (BPs), a stoichiometric basis involving nonnegative rates
• Algorithms for BPs have polynomial computational complexity scaling to system size
• BP construction illustrated for a network involving glycolysis and the TCA cycle

## Graphical abstract

**Figure:**
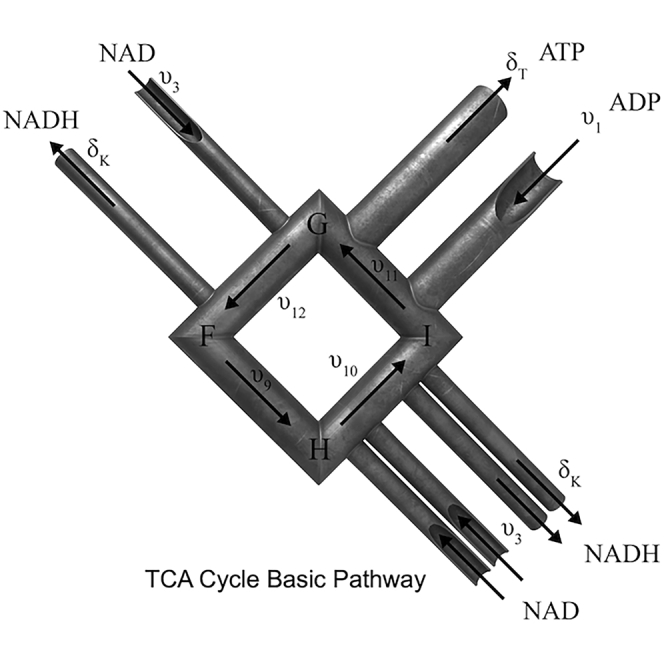

## Introduction

Even a cursory inspection of human metabolism maps reveals the astronomical complexity of the reaction networks operating within our cells. To better understand their underlying organization and structure, such networks are routinely divided into pathways, the two most popular of which are glycolysis and the citric acid (a.k.a. tricarboxylic acid or TCA) cycle. Such pathways have been defined historically and consequently with human bias; however, they also appear to follow an organizational logic, as explained in biochemistry texts. These pathways have some autonomy and independence, but they also interact with other pathways to generate interdependent network structures. An open question is whether division into these well-established pathways is the best way to decompose and comprehend such networks – or whether another approach, perhaps based on mathematical principles that are devoid of historical bias, would more effectively reveal the underlying foundational structure of such networks, assuming such structures exist.

There have been several attempts to organize biochemical reaction networks by employing mathematical principles. Three notable examples are: Schilling et al. 2000^1^ who suggested a geometric approach based upon *Extreme Pathways* defined to be the edges of the convex, polyhedral flux cone generated by the set of admissible network steady-state reaction rate vectors (a.k.a. flux modes); Schuster et al. 1994^2^ who suggested an algebraic approach based upon their notion of *Elementary Flux Modes* defined to be flux vectors having maximal sparsity; Bordbar et al. 2014^3^ who suggested a different algebraic approach based upon sparsity that required finding a linear algebraic basis of *maximum sparsity* for the set of admissible flux modes.

The extensive attention that this topic has received in the archival literature (it is clearly “well plowed turf”) leads naturally to the question of what more of interest can there possibly be to say on the subject. One of the reasons this problem continues to be a rich source of challenges to researchers is that there is no universal agreement on exactly what should constitute a definitive solution to the problem. To understand why this remains the case, consider the nature of the problem.

The biochemical metabolic network problem consists of a set of *m* metabolites subject to *n* chemical reactions. Let the *m*-dimensional, time dependent vector of metabolite concentrations be denoted by ***c****(t)* and let the *n*-dimensional vector of reaction rates be denoted by ***v****(t).* If ***S*** denotes the (constant) stoichiometric matrix corresponding to this reaction network, then the transient dynamics of the network is expressed as the dynamical system

(Equation 1) $$\dot{c}=Sv\left(t\right)$$

where ***Sv***(*t*) is understood to be the product of the matrix ***S*** and the vector ***v***(*t*). To make Equation 1 into a well-posed mathematical problem, one customarily expresses the reaction rate vector ***v****(t)* constitutively as a function of the unknown metabolite concentration vector ***c****(t)*

(Equation 2) v=f(c(t))

and then imposes an initial condition ***c****(t*_*0*_*)=****c***_*0*_ on the unknown state vector ***c****(t).* One obvious definition of “solution” to this transient metabolic network problem is to compute the solution to the dynamical system

(Equation 3) $$\dot{c}=Sf\left(c\left(t\right)\right),c\left(t_{0}\right),t>t_{0.}$$

Except for very special examples, dynamical system Equation 3 does not admit a closed, analytical solution; rather one must resort to numerical procedures to produce approximate solutions on some finite length time interval. The reaction rate constitutive relation Equation 2 usually involves many parameters most of which are poorly known and must be estimated or guessed before numerical approximations to Equation 3 can be derived. How much insight into the biochemical workings of the metabolic network one gains from computing such numerical approximations using specific parametric choices for the reaction constitutive relation is debatable.

Of particular interest in gleaning insights into a metabolic network from the dynamical system Equation 3 are investigations of qualitative issues such as the longtime behavior of solutions. Nonlinear dynamical systems Equation 3 can exhibit a wide range of longtime dynamics including convergence to a steady-state, blowup, periodic orbits and chaotic behavior. In particular, there is much interest in using dynamical system models Equation 3 to conduct *in silico* genetic knockout experiments to investigate the quantitative and qualitative effects gene mutations can have on both transient and longtime dynamics of a metabolic network. While there has been much effort given to large scale numerical simulation of transient dynamics for metabolic networks of the form Equation 3, studying their steady-state dynamics has yielded the most progress in understanding their structure and functioning.

The steady-state form of Equation 3 is

(Equation 4) Sf(c)=0.

Solving Equation 4 requires finding all steady-state metabolite vectors ***c*** satisfying the system of nonlinear algebraic Equation 4. Algebraic geometry is the mathematical discipline most closely associated with the study of such systems. While algebraic geometry is one of the most active and successful areas of modern mathematical research producing many celebrated successes, nonlinear algebraic systems Equation 4 are, in general, still poorly understood. For systems Equation 4 of even relatively modest size, answering basic questions such as how many solutions there are is extremely challenging, with only very crude bounds on the number of solutions being generally available.

For that reason, most efforts to study steady-state dynamics for metabolic networks have focused on linear systems of the form

(Equation 5) Sv=0

in which the unknown is the reaction rate vector ***v***. From Equation 5, it is evident that ***v*** is in the null-space (denoted N = Null(**S**)) of the stoichiometric matrix ***S*** but not every vector in N need be a chemically feasible reaction rate vector. In this work, chemically feasible null-space vectors ***v*** will be called *steady-state pathways* (or just pathways).

The issue of chemical feasibility for vectors in N concerns chemical reaction directionality. In particular, each of the column vectors of the stoichiometric matrix ***S*** corresponds to a particular direction of the associated reaction. If a given reaction is reversible, then the reverse direction of the reaction corresponds to the negation of the associated stoichiometric matrix column. By convention, the components of a reaction vector ***v*** corresponding to irreversible reactions are required to be nonnegative, whereas the components of ***v*** corresponding to reversible reactions can take any real value. There is no universal agreement in the literature about how best to account for reversible reactions in both the construction of the stoichiometric matrix ***S*** and the definition of chemical feasibility for its null vectors. Röhl and Bockmayr 2019^4^ give a particularly illuminating discussion of this issue. Three possible ways of resolving the ambiguity are: (1) choose a particular direction for each reversible as “positive”, let the corresponding columns of ***S*** reflect those choices, and then allow the corresponding components of permissible null-vectors ***v*** to take on any real number value; (2) augment the stoichiometric matrix ***S*** by having two columns for each reversible reaction, one for each reaction direction, and then restrict the permissible reaction vectors ***v*** to be nonnegative (Röhl and Bockmayr 2019^4^ suggest a reaction “splitting” scheme somewhat different from this but fulfilling the same purpose.); (3) for each reversible reaction, make a specific directional choice (forward or reverse), let the corresponding column of ***S*** reflect that choice and then restrict the permissible reaction vectors ***v*** to be nonnegative. As discussed below, choices (2) and (3) that restrict ***v*** to be nonnegative have certain mathematical advantages over choice (1).

As mentioned above, the definition of what solving the steady-state dynamics system Equation 5 should mean is open to interpretation. As an extreme example, suppose all reactions in the network are reversible and one adopts the first choice above for dealing with reversibility. Then all vectors in the stoichiometric null-space are chemically feasible solutions to Equation 5 and what solving Equation 5 should mean is well understood from elementary linear algebra. We know that N is a vector subspace of n-dimensional Euclidean space, and if the (m x n)-dimensional matrix ***S*** satisfies m ≤ n and Rank(***S***) = m (which is often the case for metabolic networks encountered in applications), then Dim(N) = n-m. However, just knowing that the set of all chemically feasible solutions to Equation 5 form a subspace of dimension (n-m) is not particularly useful since, unless n = m, the solution subspace consists of an infinite number of vectors. So, the phrase “solving Equation 5” is normally interpreted to mean finding a linear algebraic basis for N which is a finite set of (n-m) vectors. Even for quite large metabolic networks this is a routine task from numerical linear algebra. Suppose one denotes such a null-basis by {***n***_1_, …,***n***_r_} with r = n-m (called the *nullity* of ***S***) and then assembles these basis vectors as the columns of the (n x r)-dimensional matrix ***N*** (called a *null-basis matrix*). Different null-bases for N give rise to different null-basis matrices. A natural question is: Among the infinite number of possible null-basis matrices ***N*** that constitute a “solution” to Equation 5, are there classes of such matrices ***N*** that are more useful than others for enhancing our understanding of the metabolic network’s steady-state dynamics?

One possible answer to this question makes use of the notion of *sparsity* of ***N***. More specifically, the sparsity of ***N***, denoted Spar(***N***), is defined to be the proportion of its entries that are zero, that is,

(Equation 6) $$Spar\left(N\right)=n_{z}/\left(nr\right)$$

where n_z_ is the number of zero entries in ***N*** and nr is the total number of entries of ***N***. Clearly, Spar(***N***) is a number between 0 and 1, with Spar(***N***) = 0 meaning that none of the entries of ***N*** are zero while Spar(***N***) = 1 means ***N*** is the zero matrix. This leads to another definition of solution to Equation 5, namely, to find a null-basis matrix ***N*** of maximum sparsity, that is, Spar(***N***) is greater than or equal to the sparsity of all other null-basis matrices. This is a famous problem in mathematics and computer science called the *sparsest null-basis problem.* While finding an arbitrary null-basis is a rather routine problem of computational linear algebra, the sparsest null-basis problem is a very difficult computational challenge known to be NP-hard (meaning that its computational complexity scales faster than any power of the system’s size) (Coleman and Pothen 1986,^5^ Coleman and Pothen 1987,^6^ Gottlieb and Neylon 2016^7^). The sparsest null-basis problem arose in the setting of solving large scale optimization problems for which a popular algorithm requires finding a null-basis matrix ***N*** (Coleman and Pothen 1986^5^). The numerical efficiency of the method is strongly tied to the sparsity of ***N***. Hence, solving the sparsest null-basis problem occupies a prominent role in the original optimization problem.

Consider next the special case in which all of the metabolic network’s reactions are irreversible. In that case, a reaction rate vector ***v*** in the stoichiometric matrix null space is chemically feasible if and only if it is nonnegative, that is, ***v***≥0, and the definition of what constitutes a solution to Equation 5 must be augmented by this inequality constraint

(Equation 7) Sv=0,v≥0.

(This constraint also must hold if some of the reactions are reversible provided one employs either of the perspectives (2) or (3) for dealing with reversible reactions discussed above.).

While finding an algebraic basis for the subspace N is a routine problem in numerical linear algebra, finding all nonnegative vectors ***v*** in N is not. Indeed, it is known to be NP-hard. Interest in this problem long predates its emergence in the context of steady-state metabolic network analyses. A noteworthy early paper on the subject is Dienes 1926–1927^8^ which seems to be rarely cited by researchers in the metabolic network community. A somewhat later noteworthy contribution, also rarely cited currently, is Davis 1952^9^ which is particularly relevant to the discussion at hand, since it is evident that a geometric description of the solution to Equation 7 is that ***v*** solves the constrained system Equation 7 if and only if it lies in the intersection of the subspace N with the positive orthant of the Euclidean vector space $E^{n}$. It is straightforward to show that the geometric shape of the intersection of N with the positive orthant $E_{+}^{n}$ of $E^{n}$ is a pointed, convex, polyhedral cone denoted by $N_{+}=N\cap E_{+}^{n}$, that is generated from a finite number of extreme rays (Gerstenhaber 1951^10^). These terms will be defined precisely below, but prior to that discussion, it is worth recalling a bit of the history of these ideas.

A principal driver for the development of the theory of convex, polyhedral cones in $E^{n}$ arose from the study of various constrained optimization problems in the setting of resource allocation strategies in economics that figured prominently in confronting the extreme logistical challenges faced during the second world war. The typical situation was to optimize an objective function over a compact convex polyhedral set or an unbounded, convex polyhedral cone. This topic saw an explosion of research activity during the 1940’s culminating in a celebrated conference held at the University of Chicago in 1949 with an associated conference proceedings Koopmans 1951^11^ containing what are now widely viewed as the seminal presentations of the foundational theories of several aspects of the central resource allocation problem that gave rise to such subjects as linear programming, convex analysis, convex polyhedral cones, etc. The chapters Gale 1951^12^ and Gerstenhaber 1951^10^ from Koopmans 1951^11^ are of particular importance to the subject at hand in this paper as are the related papers by Davis 1952,^9^ 1953,^13^ 1954^14^ that were strongly motivated by Gale 1951^12^ and Gerstenhaber 1951.^10^

To facilitate the discussion, it proves helpful to fix notation and several definitions. Given two sets *A* and *B* in $E^{n}$, their sum *A+B* is defined to be the set

(Equation 8) A+B={a+b|foralla∈A,b∈B}.

The smallest subspace containing *A* is denoted by D{*A*} and called the *dimensionality space of A*. Clearly, D{*A*}is the intersection of all subspaces in $E^{n}$ containing *A.* The dimension of D{*A*}is called the *dimension of A* and denoted d{*A*}. For given nonzero ***a***∈ $E^{n}$, the set (***a***) = {λ***a*** | for all λ ≥ 0} is called a *half-line* or a *ray*. The positive orthant of $E^{n}$ is denoted by $E_{+}^{n}$. The vector ***a***∈ $E^{n}$ is a *strictly positive linear combination* of the vectors ***a***_1_, …,***a***_k_ provided ***a*** = λ_1_ ***a***_1_, …, λ_k_ ***a***_k_ for λ_1_, …,λ_k_ > 0, and it is called a *strictly convex linear combination* if in addition λ_1_+ … +λ_k_ = 1. It is evident from these definitions that the set of all strictly convex linear combinations of two distinct points ***a*** and ***b*** is the open line segment connecting the two points. A set *S* in $E^{n}$ is called *convex* provided for every pair of points in *S*, the line segment joining the two points is also in *S*. Given a set of points *A* in $E^{n}$, its *convex hull* is defined to be the intersection of all convex sets containing *A.* The convex hull of all subspaces contained in *A* is called the *lineality space of A* and denoted L{*A*}; its dimension, denoted l{*A*}, is called the *lineality of A.* A *convex cone* in $E^{n}$ is defined to be the convex hull of a set of rays, while a *convex polyhedral cone* is the convex hull of a *finite* number of rays. For a given point ***a*** in a convex cone, (***a***) is an *extreme ray* of the convex cone if and only if ***a*** is not a positive linear combination of any two distinct points in *A* different from ***a***. The convex hull of rays (***a***_1_), …,(***a***_r_) is the set of all positive linear combinations of ***a***_1_, …,***a***_r_ which is (***a***_1_)+ … +(***a***_r_). A *frame* of a convex polyhedral cone is a finite set of rays which span (using only nonnegative linear combinations) the cone and such that no ray of the set is in the convex hull of the others. A convex polyhedral cone *C* is called *pointed* if there exists a half-space that intersects the cone only at the origin. A pointed convex polyhedral cone is spanned by its extreme rays, that is, *C =* (***c***_1_)+ … +(***c***_r_), where (***c***_1_), …,(***c***_r_) are its extreme rays. A convex polyhedral cone is pointed if and only if l{*C*} = 0, that is, its lineality is zero. A convex polyhedral cone *C* in $E^{n}$ is called *solid* if D{*C*} = $E^{n}$, that is, the smallest subspace containing *C* is $E^{n}$.

From these results provided by Gerstenhaber 1951,^10^ Gale 1951^12^ and Davis 1952,^9^ 1953,^13^ 1954,^14^ one gleans that all solutions to the constrained steady-state system Equation 7 lie in the pointed, convex, polyhedral flux cone $N_{+}=N\cap E_{+}^{n}$ whose extreme pathways form a generating set for N_+_. Hence, an appealing definition of a solution to the constrained steady state system Equation 7 would be to calculate all of the extreme pathways of N_+_. As compelling as this logic might seem, a major obstruction to the use of this definition of a solution to Equation 7 is that the problem of calculating all extreme pathways of N_+_ is of computational complexity NP hard. Moreover, the number of extreme pathways, in general, grows exponentially with system size *nm*. Even for the most effective algorithms currently available in the literature for computing extreme pathways, as pointed out in Röhl and Bockmayr 2019,^4^ for example, it is not hard to find metabolic networks for which the algorithms fail to produce a complete list of extreme pathways. Also, even for moderately sized networks with *nm* $∼10^{2}-10^{3}$*,* the number of extreme pathways can exceed $10^{4}-10^{5}$ making it a daunting if not impossible task to ascertain the chemical or biological significance of each extreme pathway.

If some (or all) of the system’s reactions can be viewed as reversible, then possibly different definitions of what constitutes a solution to the steady-state reaction network system must be utilized. Three possible strategies for dealing with reversible reactions were described above, the last two of which resulted in consideration of a pointed flux cone $N_{+}=N\cap E_{+}^{n}$ satisfying the constrained steady-state system Equation 7 allowing one to define a solution, as in the case of all reactions being irreversible, to be finding the set of extreme pathways of $N_{+}$. The first strategy requires that the reversible reactions be permitted to have both positive and negative flux rates. This greatly complicates the structure of the metabolic flux cone as shown in Schuster and Hilgetag 1994^2^ and Röhl and Bockmayr 2019^4^ and is discussed below.

The discussion of the first model for handling reversibility is facilitated by introducing some notation. Consider the reaction rate index set *Ind = {1, …,n}* and define the two subsets *V*_*irr*_ and *V*_*rev*_ by: *V*_*irr*_ denotes the subset of *Ind* consisting of the indices corresponding to the irreversible reaction rates and *V*_*rev*_ denotes the complementary subset of *Ind* consisting of the indices corresponding to the reversible reactions. Let |*V*_*irr*_| and |*V*_*rev*_| denoted the cardinality of *V*_*irr*_ and *V*_*rev*_, respectively. The constrained steady-state system Equation 7 is replaced by the constrained system:

(Equation 9) $$Sv=0,v_{j}\geq 0\;for\;j\in I_{irr}\text{.}$$

It is straightforward to see that the set of pathways ***v*** satisfying Equation 9 form a convex polyhedral cone C in $E^{n}$. If C is pointed, then it is generated by its extreme pathways (edges). However, C need not be pointed. It is known from Gerstenhaber 1951^10^ that the convex polyhedral cone C is pointed if and only if its lineality l{C} is zero, that is, {***0***} is the only subspace of $E^{n}$ contained in C. How this is connected to reaction reversibility is straightforward. An individual reaction of the system is called reversible if its reaction rate can take both positive and negative values. A pathway ***v*** satisfying Equation 9 is called reversible if -***v*** also satisfies Equation 9. Clearly then, a pathway vector ***v*** is reversible if and only if all of its non-zero component reaction rates correspond to reversible reactions, and a metabolic network has a pointed flux cone of solutions two Equation 9 if and only if it has no reversible pathways ***v*** even though some of its individual reactions can be reversible. In summary, if a metabolic network has no reversible reactions, then its steady state convex polyhedral flux cone N is pointed and all of its extreme rays lie in the positive orthant $E_{+}^{n}$, whereas if it has reversible reactions but no reversible steady state pathways, then its steady state flux cone C is pointed but its extreme rays do not all lie in $E_{+}^{n}$.

The question now arises: How can a frame generating the convex polyhedral cone C of solutions to Equation 9 be found if C is not pointed, that is, if its lineality l{C} is non-zero? It is shown in Gerstenhaber 1951^10^ that C must have a finite frame of rays {(***a***_*1*_), …,(***a***_*k*_)} and that the lineality subspace *L*{C} of C is the convex hull of the frame rays (***a***_*j*_) contained in *L*{C}. While finding a frame for C might seem like a reasonable definition of a solution to Equation 9 since it provides a generating set for C of minimal cardinality, in general, it is a challenging problem of NP hard computational complexity.

As an alternative to finding a frame for a non-pointed convex polyhedral cone C of solutions to Equation 9, Schuster and Hilgetag 1994^2^ introduce the notion of *Elementary Flux Modes* for C. An elementary flux mode is an inclusion-minimal pathway ***v*** satisfying Equation 9. It is shown in Schuster and Hilgetag 1994^2^ that the extreme rays of C, if any, are all elementary flux modes and that if C is pointed, then the set of elementary flux modes and the set of extreme rays coincide. In practice, the cardinality of the set of elementary flux modes in C is much larger than the cardinality of a frame for C for large metabolic networks, and finding all of the elementary flux modes of C is computational task of NP hard complexity.

Due to the extreme redundancy of using the set of elementary flux modes as a generating set for C, Röhl and Bockmayr 2019^4^ introduce the notion of a *MEMo,* an inclusion minimal set of elementary flux modes that form a set of generators for C. It should be noted that a *MEMo*, as defined by Röhl and Bockmayr 2019^4^ is not a frame as defined by Gerstenhaber 1951.^10^ Both a *MEMo* and a frame provide a generating set for C, but for a frame, linear combinations must use only nonnegative coefficients while for a *MEMo*, negative coefficients are permitted for reversible pathways in the *MEMo.*

Bordbar et al. 2014^4^ introduce an algorithm they call MinSpan that they conclude produces a stoichiometric null-basis of maximum sparsity in which each pathway satisfies certain inequality constraints. In effect, they argue that having a null-basis with maximum sparsity enhances our understanding of the full network’s steady-state dynamics and should be considered as defining a solution to Equation 5. However, the authors do not provide a proof that their MinSpan algorithm does what they claim, but seem to suggest, without justification, that a proof contained in Coleman and Pothen 1986^5^ will also prove that MinSpan converges to a sparsest stoichiometric null-basis subject to unspecified “thermodynamic constraints”. Nor do they mention that the MinSpan algorithm must be NP-hard to implement. In particular, it is easy to see that the key portion of the MinSpan algorithm contained in the rectangular box of page 12 of Bordbar et al. 2014^4^ is NP-hard. Additionally, in the authors’ description of the MinSpan algorithm, the proof they present on page 12 is flawed. In their proof that the vector ***x*** is linearly independent of the columns of the matrix ***P****′,* the authors introduce the matrix $\Theta =N^{-1}P^{\prime }$, but the matrix **N** is, in general, not invertible or even a square matrix. This invalidates their proof. Nor do the authors demonstrate that the MinSpan basis provides an acceptable definition of a solution to the dynamic, steady-state metabolic network problem. As discussed in the introductory section of this paper, any meaningful definition of a solution to this problem must specify a finite generating set of fluxes by which every admissible flux vector in the stoichiometric null-space can be constructed as a linear combination of vectors in the generating set whose coefficients can be computed by an easily implemented algorithm. It seems that the authors intended for their MinSpan basis to be the required generating set for admissible fluxes. It is also clear that the set of admissible fluxes is defined to be all vectors in the stoichiometric null-space satisfying “… biological and thermodynamic constraints (lb and ub) …” with lb and ub being vectors of lower and upper bounds on generic flux vectors. The physical nature of these biological and thermodynamic constraints is not discussed. As a result of these lower and upper bounds, the set of admissible fluxes in their model comprise a *compact* convex polyhedral subset of the stoichiometric null-space. The issue of whether or not their MinSpan basis provides a useful generating set for the compact set of admissible fluxes was not discussed. The issue is specifying necessary and sufficient conditions on coefficients (which can take both positive and negative values) in linear combinations of the MinSpan base vectors that produce flux vectors satisfying the lower and upper vector bounds required for admissible fluxes.

The present contribution defines a solution to the constrained steady state system Equation 7 that differs from the various definitions (set of extreme pathways, set of elementary flux modes, MEMo, MinSpan) just discussed. In particular, its computational complexity avoids the “curse of dimensionality” inherent in all of the other solution definitions. The definition of solution utilized in this work requires finding an n×(n−m) dimensional null-basis matrix, ***W***, for an m×n dimensional stoichiometric matrix ***S*** (of rank *m* with *n>m*) consisting of column vectors ***BP***_*j*_*, j=1, …,*(*n-m*), called *basic pathways,* each of which satisfies the nonnegativity inequality constraint in Equation 7. The salient features of this approach to solving Equation 7 are as follows.

(1) The metabolic network is assumed to be occurring in a cell that is experiencing steady exponential growth resulting in a steady exponential rate of decline in metabolite concentrations (This assumption is generalized below.). It is frequently argued that cell growth can be ignored in analyzing metabolic networks since the rates of internal and external reactions are usually much larger than the dilution rates associated with cell growth (Horvat et al. 2015^15^). While this argument often has merit for short term transient dynamic analyses, such is not the case for steady state analyses. It is shown that the dilution effects, when incorporated into the stoichiometric matrix, facilitate the direct construction of an n×(n−m) dimensional null basis matrix ***G*** for ***S*** whose columns form a *Fundamental Null Basis* as defined by Coleman and Pothen 1987^6^ to meaning that ***G*** has an imbedded identity matrix ***I***_*(n-m)*_ of maximum rank. The matrix ***G*** has high sparsity though not necessarily maximum sparsity and, in general, its non-zero entries are not all positive. A Gauss-elimination-inspired algorithm is then employed to transform ***G*** into ***W*** whose non-zero entries are all positive. A tool called *Singleton Theory* is developed to guide this transformation of ***G*** into ***W*** in a way that maintains high sparsity. If ***W*** has a property called *Singleton Complete,* it is proved that its columns coincide with the set of extreme pathways of ***S*.**
(2) While the basic pathways ***BP***_*j*_*, j=1, …,*(*n-m*) do not necessarily form a frame for the pointed convex polyhedral flux cone $N_{+}$, they do span it in the linear algebraic sense, that is, allowing linear combinations to have some negative coefficients. However, appealing to singleton theory, easily computable necessary and sufficient inequality conditions are derived on any negative coefficients to ensure that a pathway ***v*** represented as a linear combination of the basic pathways satisfies Equation 7, that is, lies in $N_{+}$.
(3) Singleton theory shows how the components of a general pathway ***v*** in $N_{+}$ can be decomposed into two sets: (1) independent parameters and (2) dependent parameters with readily computed closed form algebraic functions by which values for the dependent parameters are determined from any given choice of values for the independent parameters.
(4) *Basic pathways* are defined to span the steady state flux cone $N_{+}$ corresponding to the stoichiometric matrix for a metabolic network within a steadily growing cell. Singleton theory provides key tools for proving the central properties of a basic pathway null basis contained in the flux cone $N_{+}$.
(5) *Basic pathway* theory is illustrated using a metabolic network inspired by the one developed by Bordbar et al. 2014,^3^ augmented by the inclusion of dilution reactions that account for cell growth effects. Finally, the significance of these results is discussed and conclusions are drawn.

Other influential works have addressed these topics. Certainly, the seminal contribution Clarke 1988^16^ must be acknowledged. Also, it behooves us to cite algorithmic work done on finding frames for pointed conical polyhedral sets (Dulá et al. 1998,^17^ 2006;^18^ Wets et al. 1967,^19^ 1968^20^) and the more abstract results of Kuhn 1956^21^ since these provide theoretical underpinnings to algorithmic development directed to finding extreme pathways and elementary modes for biochemical networks. In that setting, the important work of Klamt et al. 2002^22^ and 2006,^23^ Schuster et al. 1993^24^ and 2002,^25^ Llaneras et al. 2010,^26^ Papin et al. 2004,^27^ Wiback et al. 2003,^28^ Yanping et al. 2019,^29^ and Yeung et al. 2007^30^ should be mentioned. Finally, work on exploring ways of dealing with the curse of dimensionality inherent in extreme pathway, elementary flux modes and minimal spanning sets by Jevremovic et al. 2010,^31^ 2011,^32^ 2011,^33^ 2013^34^ is germane to the issues of interest here.

## Results

Subsection Model formulation formulates the model to be studied corresponding to steady-state metabolic dynamics within a cell undergoing steady exponential growth while Subsection The stoichiometric null-space describes the special structure the associated stoichiometric null-space has due to cell growth. Subsection Definition of basic pathways is devoted to construction of the ***W*** matrix whose column vectors, called *basic pathways,* form a linear basis for the stoichiometric null-space. Subsection Singleton theory presents the *singleton theory* of matrices that contains essential tools for using the basic pathways as a fundamental mechanism for understanding steady-state network dynamics. Subsection Singleton cost describes *singleton cost*. Finally, Subsection Case study is devoted to a case study used to illustrate the power of the basic pathways approach.

### Model formulation

Consider a metabolic reaction network consisting of *m* chemical components subject to *r* internal reactions inside a cell that is growing at a steady exponential rate α. The exponential growth rate of the cell gives rise to a dilution rate α*[C*_*i*_*]* for each chemical component *C*_*i*_ where *[C*_*i*_*]* denotes the concentration of the chemical component *C*_*i*_. The system’s *m x n* stoichiometric matrix *S,* with *n* = *m*+*r*, can be given the structure

(Equation 10) $$S=\left(S_{0}/{-I}_{m}\right)$$

where ***I***_*m*_ denotes the *m*×*m* identity matrix (ones down the main diagonal and zeros off the main diagonal) accounting for the growth induced dilution effects and ***S***_***0***_ denotes the *m*×*r* stoichiometric sub-matrix accounting for the internal reactions.

### The stoichiometric null-space

Substitution of Equation 10 into Equation 5 gives the homogeneous linear system

(Equation 11) $$Sv=S_{0}v_{0}-v_{1}=0$$

where the n-dimensional vector ***v*** has the form

(Equation 12) $$v=\left(\begin{array}{c} v_{0}\\ \cdots \\ v_{0} \end{array}\right)$$

with ***v***_0_ and ***v***_1_ being r-dimensional and m-dimensional vectors, respectively. It follows from Equations 11 and 12 that ***v*** is in the null space of ***S*** if and only if

(Equation 13) $$S_{0}v_{0}=v_{1}\text{,}$$

and hence that the stoichiometric null space consists of all vectors of the form Equation 12 with ***v***_0_ being an arbitrary r-dimensional vector and ***v***_1_ specified by Equation 13. A basis for the null-space of ***S*** can be readily constructed by sequentially letting ***v***_0_ represent each of the natural basis vectors of the *r*-dimensional Euclidea. n vector space and using Equation 13 to calculate the corresponding m-vector ***v***_1_. This sequential process can be combined into the matrix calculation

(Equation 14) $$V_{1}=S_{0}I_{r}=S_{0}\text{.}$$

From Equation 14, it follows that a basis for the r-dimensional null-space of ***S*** can be written as the columns of the *n x r -* dimensional matrix

(Equation 15) $$G=\left(\begin{array}{c} I_{r}\\ \ldots \\ S_{0} \end{array}\right)=\left(g_{1},\ldots ,g_{r}\right)\text{.}$$

### Definition of *basic pathways*

Assume that the chemical admissibility criterion for a stoichiometric null-vector ***v*** is nonnegativity. In general, not all of the column vectors ***g***_1_, … , ***g***_r_ in Equation 15 need be nonnegative. In particular, while the first r-rows of the matrix ***G*** in Equation 15 have only nonnegative entries, the submatrix ***S***_0_ may have negative entries. Notice that an algebraically equivalent null-space basis matrix can be constructed by applying to Equation 15 a sequence of the three basic column operations: (1) multiply a column of Equation 15 by a non-zero scalar, (2) interchange two columns of Equation 15 or (3) replace a column of Equation 15 by the sum of it and a non-zero scalar multiple of another column.

The first *r*-rows remain nonnegative if and only if positive scalars are used in the type (1) and type (3) column operations. Assume first that each row of ***S***_*0*_ contains at least one positive entry. How to proceed if ***S***_*0*_ contains rows without a positive entry will be considered subsequently. Assuming that every row of ***S***_*0*_ has at least one positive entry allows an algebraically equivalent null-space basis matrix for Equation 15 to be constructed in which all entries are nonnegative. This matrix can be generated by applying a succession of column operations of type (3) starting from the bottom (i.e., row *n*) of Equation 15 and sequentially moving upward to row (*r+1*). The resulting nonnegative null-space basis matrix (in column form) will be designated

(Equation 16) $$W=\left({BP}_{1},\ldots ,{BP}_{r}\right)\text{.}$$

Thus, every column of ***W****,* as well as any linear combination of columns using positive coefficients, is a chemically feasible stoichiometric null space reaction rate vector. The column vectors of ***W*** are called *basic pathways* in this work. Since the columns of ***W*** constitute a set of basis vectors for the null space of ***S***, all chemically relevant (i.e., nonnegative) null space vectors can be constructed as a linear combination of those basis vectors Equation 16. In this work, the term *pathway* refers to any nonnegative (chemically feasible) vector ***v*** contained in the stoichiometric null space. Hence, every *pathway* ***v*** has a unique representation

(Equation 17) $$v=r_{1}{BP}_{1}+\ldots +r_{r}{BP}_{r}$$

Assume next that the matrix ***S***_*0*_ contains one of more rows without a positive entry. More specifically, suppose that row *k* of ***S***_*0*_ has no positive entries and that it has *q* entries that are negative with the remaining *r-q* entries being zero. For convenience, rearrange the columns of ***S***_*0*_ so that the first *q* entries of row *k* are negative with the subsequent *r-q* entries begin zero. For this ***S***_*0*_, a nonnegative pathway ***v****=(v*_*j*_*)* satisfies Equations 5 and 10 only if

(Equation 18) $$v_{1}=v_{2}=\ldots =v_{q}=v_{\left(r+k\right)}=0$$

Notice that Equation 18 implies that columns 1, 2, …,q of ***S***_*0*_ play no role in solving the steady-state Equation 5. Hence, one can modify ***S***_*0*_ by removing its first *q* columns and its *k*^th^ row producing the reduced *(m-1)*x*(r-q)-*dimensional matrix ***S***_*R0*_ and corresponding ***S***_*R*_*= (****S***_*R0*_ |***I***_*(m-1)*_*),* and conclude that the positive entries in any *n*-dimensional pathway vector ***v*** satisfying Equations 5 and 10 are contained in the corresponding *(n-q-1)*-dimensional vector ***v***_*R*_ satisfying

(Equation 19) $$S_{R}\upsilon_{R}=0$$

Since the steady-state dilution rate corresponding to the metabolic component *C*_*k*_ must be zero, the concentration [*C*_*k*_] of *C*_*k*_ must approach zero at steady-state.

Generalizing this argument to arbitrary *m*x*r-*dimensional matrices ***S***_*0*_, to find all nonnegative *n*-dimensional pathway vectors ***v*** satisfying Equations 5 and 10 one can find all non-zero entries of ***v*** by first removing from ***S***_*0*_ all of its rows that do not contain at least one positive entry and removing all of its columns that contain a non-zero entry in at least one of the removed rows. The remaining reduced matrix ***S***_*R0*_ has the property that all of its rows have at least one positive entry and all of the non-zero entries in every *n-*dimensional nonnegative pathway vector ***v*** satisfying the original system Equations 5 and 10 are non-zero entries in the reduced nonnegative pathway vector solutions ***v***_*R*_ satisfying the reduced system Equation 19. For an extreme case, suppose that no rows of ***S***_*0*_ contain a positive entry. Then clearly the only nonnegative pathway vector ***v*** satisfying Equations 5 and 9 is ***v****=****0*** and the concentrations of all metabolic components must vanish at steady-state.

Returning to the most interesting case that every row of ***S***_*0*_ contains a positive entry, the basic pathways Equation 16 form an algebraic basis for the *r-*dimensional null-space of the stoichiometric matrix ***S,*** denoted by N. Clearly, the basic pathways cannot constitute a *frame* for the stoichiometric null-space since linear combinations Equation 17 in which all of the coefficients r_*1*_*, …,r*_*r*_ are nonnegative yield pathway vectors lying in the positive orthant $E_{+}^{r}$ of *r*-dimensional Euclidean space $E^{r}$. Recalling from the introductory section and Davis, 1952,^9^ 1953,^13^ 1954^14^ that the nonnegative pathways all lie in a pointed convex polyhedral cone $N_{+}=N\cap E_{+}^{r}$ contained in $E_{+}^{r}$, the question naturally arises: Under what conditions do the basic pathways form a frame for $N_{+}$? From Gerstenhaber, 1951^10^ and Davis, 1952,^9^ 1953^13^ one knows that the extreme vectors in $N_{+}$ form a frame which leads to the question: Under what conditions are the basic pathways extreme vectors of $N_{+}$? This question can be addressed by *singleton theory* discussed in the following subsection.

### Singleton theory

Let ***M*** be an *n*x*r*-dimensional matrix with *0*≤*n-r*. Let the components of ***M*** be given by ***M****=[m*_*ij*_*]* for i*=1, …,n* and *j=1, …,r.* Let the column vectors of ***M*** be denoted by ***M****=[****c***_*1*_*, …,****c***_***r***_*].* Row *i* of ***M*** is said to be a *singleton row* if it contains only one non-zero entry. Column ***c***_*j*_ is said to have a *singleton element m*_*ij*_ if row *i* is a singleton row with *m*_*ij*_ being its only non-zero entry. The matrix ***M*** is said to be *singleton complete* if every column ***c***_*j*_ for *j=1, …,r* has at least one singleton element. If one or more columns of ***M*** does not contain a singleton element, then ***M*** is called *singleton deficient.* The number of singleton rows in ***M*** is called its *singleton row rank* while the number of singleton deficient columns of ***M*** is called its *singleton deficiency index.* We apply these ideas to the basic pathways matrix ***W*** [16] in the following theorem.

**Theorem 2.1** If the basic pathways matrix ***W*** Equation 16 is singleton complete, then the reaction vector ***v*** given by Equation 17 is nonnegative if and only if all of the reaction rate coefficients *r*_*j*_, for *j=1, …,r*, are nonnegative.

**Proof:** The “if” portion of the theorem is obvious since each of the basic pathway vectors ***BP***_*j*_*, j=1, …,r* is in the cone $N_{+}$**,** and hence is nonnegative, implying that the representation Equation 17 must also be nonnegative if all of the rate coefficients *r*_*j*_*,* for *j=1, …,r* are nonnegative. For the “only if” portion of the theorem, it is helpful to introduce notation for the entries in the matrix ***W****=[w*_*ij*_*], i=1, …,n, j=1, …,r.* By assumption, each basic pathway vector ***BP***_*j*_ has at least one singleton component. Let *i(j)* denote the row index of a singleton component of ***BP***_*j*_ for each *j,1, …,r*. Then from Equation 17 and the fact that *w*_*i(j)j*_ is a singleton component of ***BP***_*j*_, it follows that the *i(j)*^th^ component of ***p****=[v*_*j*_*]* is given by

(Equation 20) $$v_{i\left(j\right)}=r_{j}w_{i\left(j\right)j}\text{.}$$

Since *w*_*i(j)j*_ is nonnegative, it follows that *p*_i(j)_ is nonnegative only if *r*_*j*_ is nonnegative. Since Equation 20 must hold for each *j=1, …,r,* one concludes that ***v*** given by Equation 17 is nonnegative only if each *r*_*j*_, for *j=1, …,r*, is nonnegative, thereby proving the theorem. □

Observation Equation 20 shows that for a basic pathways matrix ***W****,* one knows the mixing coefficients *r*_*j*_*,* for *j=1, …,r* in Equation 17 if and only if one knows the pathway components *v*_i(j)_*,* for *j=1, …,r.* For the task of finding *r*_*j*_*,* for *j=1, …,r*, in Equation 16, one can divide the components of ***v*** into the two groups, *v*_i(j)_ for *j=1, …,r*, and the remaining *m=n-r* components. The components *v*_i(j)_*,* for *j=1, …,r,* will be considered as free parameters while the remaining *m* will be viewed as dependent parameters. Hence, if the free parameters *p*_i(j)_*,* for *j=1, …,r,* are specified, then the mixing coefficients *r*_*j*_*,* for *j=1, …,r,* are given by Equation 20 and the now known right-hand-side of Equation 17 can be viewed as determining the *m* dependent components of ***v***. Thus, the *m* dependent components of ***v*** are given as functions of the *r* independent components of ***v***. These functions will be viewed as *compatibility relations* among the components of ***v*** enforcing the steady-state condition

(Equation 21) Sυ=0

Since the basic pathways form an algebraic basis for the stoichiometric null-space N, it follows that none of the basic pathways can be written as a positive linear combination of the others. Appealing to Gerstenhaber 1951,^10^ one can then conclude that the basic pathways *{****BP***_*j*_, *j=1, …,r}* are the extreme vectors of $N_{+}$.

**Theorem 2.2** For the pointed convex polyhedral cone $N_{+}=N\cap E_{+}^{r}$, the set of basic pathways ***{BP***_*j*_**,** *j=1, …,r****}*** coincides with the set of extreme pathways.

**Proof:** According to Gerstenhaber, 1951,^10^ a vector ***d*** in $N_{+}$ is an extreme vector provided it cannot be written as a convex combination of two distinct vectors in $N_{+}$. That is, it is impossible to write

(Equation 22) $$d=Rv_{1}+\left(1-R\right)v_{2}$$

with ***v***_*1*_*,* ***v***_*2*_ being distinct vectors in $N_{+}$ and *0<R < 1.* Also, if ***d*** is an extreme vector of $N_{+}$*,* then all of the non-zero vectors in the infinite ray *(****d****)* generated by ***d*** (the set of all nonnegative scalar multiples of ***d***) are also extreme vectors. Suppose that ***d*** is not contained in any of the infinite rays generated by the basic pathway vectors ***BP***_***j***_***,*** j = *1, …,r*. Then ***d*** can be written as a positive linear combination of the basic pathways ***d*** = r_*1*_***BP***_*1*_*+ … +r*_*r*_***BP***_*r*_ with at least two of the coefficients being non-zero. Suppose that r_*k*_ > 0. Then ***d*** can be written as

(Equation 23) $$d=r_{k}{BP}_{k}+c,c=r_{1}{BP}_{1}+\ldots +r_{\left(k-1\right)}{BP}_{r\left(k-1\right)}+r_{\left(k+1\right)}{BP}_{\left(k+1\right)}+\ldots +r_{r}{BP}_{r}$$

After obvious rearrangement, Equation 23 takes the form Equation 22 with

(Equation 24) $$R=\frac{r_{k}}{1+r_{k}},v_{1}=\left(1+r_{k}\right){BP}_{k},v_{2}=\left(1+r_{k}\right)\;c$$

which contradicts the assumption that ***d*** is an extreme vector for $N_{+}$***.*** Hence, ***d*** must be a scalar multiple of one of the basic pathways ***BP***_*j*_*,* j = *1, …,r*. On the other hand, since the set of extreme pathways and the set of basic pathways form generator sets for $N_{+}$, they must produce the same set of infinite rays and hence have the same cardinality thereby proving the theorem.

Two important questions arise in applying these singleton concepts to studying representations of the pointed convex polyhedral cone $N_{+}$: First, since ***G*** Equation 15 is obviously singleton complete and ***W*** is constructed from ***G*** via a Gauss-elimination-like process, can one guide the process so that it leads to a singleton complete ***W*** or minimizes ***W***’s singleton deficiency index? Second, when ***W*** is singleton deficient, how can the chemically feasible pathways ***p*** of the form ***v*** *= r*_*1*_***BP***_*1*_*+ … +r*_*r*_***BP***_*r*_ *=* ***W***⋅***r*** *> 0,* where ***r*** denote the r-dimensional vector of pathway rates *{r*_*1*_*, …,r*_*r*_*}*, be characterized?

To answer these questions, it proves useful to introduce additional terminology. Consider a generic matrix ***M*** whose columns form a basis for the stoichiometric matrix null space. The process of transforming matrix ***G*** into the basic pathways matrix ***W*** creates a sequence of such matrices ***M***_*1*_, ***M***_*2*_, … etc. The matrix ***G*** is obviously singleton complete, since its top *r* rows are the *r*-dimensional identity matrix ***I***_*r*_, but the matrix ***W*** need not be. What is needed is an understanding of how the process of transforming ***G*** into ***W*** affects the singleton properties of the sequence of intermediate matrices ***M***, in particular, their singleton row rank and their singleton deficiency index. It is important to note that the singleton row rank and the singleton deficiency index are logically independent concepts. Thus, for example, it is possible for a matrix to undergo a transformation that increases its singleton row rank while also increasing its singleton deficiency index.

The process by which the basis matrix ***G*** is transformed into the basic pathways matrix ***W*** consists of sequentially applying three elementary column operations to ***G*** and successive matrices ***M***. A Type-(i) elementary column operation on ***M*** or ***G*** consists of multiplying a column of ***M*** by a positive number. A Type-(ii) elementary operation consists of interchanging two columns of ***M***, while a Type-(iii) elementary column operation consists of replacing a column of ***M*** by a positive linear combination of it and another column on ***M***. Clearly, these operations insure that the columns of the successive matrices ***M*** arising during the transformation process form a basis for the stoichiometric null space and that the upper *r×r* submatrix of ***M*** has only nonnegative entries. Through use of these column operations, all the negative entries in ***G*** can be removed resulting in the basic pathways matrix ***W***. The issue that remains to understand is how use of these column operations affects the singleton row index and the singleton deficiency index of ***G***.

To that end, it is useful to introduce the concept of *singleton cost* of applying an elementary column operation to a matrix. A *Type-I singleton cost* is defined to be the change in the singleton row rank resulting from application of the column operation whereas a *Type-II singleton cost* is defined to be the change in the singleton deficiency index. By convention, if application of a column operation reduces the singleton row rank of a matrix, the Type-I singleton cost of that operation is taken to be positive; correspondingly, if the singleton row rank of the matrix is increased, the Type-I singleton cost of the operation is taken to be negative. Similarly, the Type-II singleton cost of a column operation is positive if the singleton deficiency index increases while it is negative if the singleton deficiency index decreases. It is straightforward to show that column operations of types one and two change neither the singleton row index nor the singleton deficiency index of a matrix upon which they are applied and therefore have singleton cost zero. In contrast, column operations of type three can have positive, zero or negative singleton cost of both types.

### Singleton cost

The following describes how the application of these three types of column operations affect singleton costs.

1. Consider a Type-(iii) elementary operation of the form: column ***c***_*j*_ is replaced by the sum ***c***_*j*_ + *a****c***_*k*_ where *a* is a non-zero scalar and *j* and *k* are distinct indices. If the column vector ***c***_*k*_ contains no singleton entries, then the singleton cost of this Type-(iii) operation is zero. If ***c***_*k*_ contains singleton entries, then the Type-(iii) operation ***c***_*j*_ + *a****c***_*k*_ generally carries a singleton cost as illustrated in the following example.

For a column vector ***c***_*k*_, let its entries be denoted (*c*_*ik*_). Suppose that ***c***_*k*_ contains a single singleton entry *c*_*ik*_ and that application of the Type-(iii) column operation ***c***_*j*_ + *a****c***_*k*_ produces no new singleton rows. Then the singleton cost of ***c***_*j*_ + *a****c***_*k*_ is one since the entry *c*_*ik*_ is no longer a singleton entry of the *i*^*th*^ row thereby reducing the singleton row rank by one.

2. The strategy utilized in the selection of a sequence of elementary column operations applied to a stoichiometric null space basis matrix ***G*** of the form Equation 15 in order to produce the basic pathways matrix ***W*** Equation 16 is to minimize net singleton cost. The objective is to devise such a sequence that results in a basic pathways matrix ***W*** Equation 16 that is also singleton complete or has minimal singleton deficiency index. In general, this task is facilitated by minimizing the singleton cost of each type three column operation applied in the sequence of elementary column operations used to transform ***G*** into ***W*.**

The second question posed above concerns the complete characterization of the sets of rate coefficients *{r*_*1*_*, …,r*_*r*_*}* in Equation 17 that guarantee the pathway ***v*** to be chemically feasible, i.e., nonnegative. When ***W*** has singleton deficiency index zero, then the answer is trivial, ***v*** in Equation 17 is nonnegative if and only if all the rate coefficients are nonnegative. As shown below, when ***W*** has singleton deficiency index greater than zero, then a complete characterization of the rate coefficients *{r*_*1*_*, …,r*_*r*_*}* in Equation 16 giving rise to nonnegative pathways ***v*** can be constructed in the form of lower bounds on the rate coefficients that are necessary and sufficient to guarantee the nonnegativity of ***v***. The construction and its proof are given inductively on the singleton deficiency index of ***W***. Thus, the first case considered is when ***W*** has singleton deficiency index one. Without loss of generality, we can always rearrange the list of basic pathway vectors so that ***BP***_*1*_ is singleton deficient while ***BP***_*2*_*, …,****BP***_*r*_ are singleton complete, that is, each possesses at least one singleton entry. It follows that in order for ***v*** in Equation 17 to be nonnegative it is necessary for *r*_*2*_*, …,r*_*r*_ to all be nonnegative. More explicitly, we desire satisfaction of the vector inequality

(Equation 25) $$0<v=r_{1}{BP}_{1}+r_{2}{BP}_{2}+\ldots +r_{r}{BP}_{r}$$

which is shorthand notation for the system of inequalities

(Equation 26) $$0<r_{1}w_{i1}+r_{2}w_{i2}+\ldots +r_{r}w_{ir}for\;i=1,\ldots ,n\text{.}$$

We know that *r*_*2*_*, …,r*_*r*_ must all be nonnegative. The only interesting case of Equation 26 is when *r*_*1*_ *< 0* which is then equivalent to the system of inequalities

(Equation 27) $$\left|r_{1}\right|w_{i1}<r_{2}w_{i2}+\ldots +r_{r}w_{ir}\;for\;i=1,\ldots ,n\text{.}$$

The inequalities Equation 27 are only non-trivial for those row index values *i* for which *w*_*i1*_ *>0* in which case Equation 27 is equivalent to

(Equation 28) $$r_{1}>-\min\left\{r_{2}{wi}_{2}+\ldots +r_{r}w_{ir}/w_{i1}\right\}$$

where the minimum in Equation 28 is taken over all row index values *i* for which *w*_*i1*_ *>0.* Thus we see that Equation 26 holds for ***W*** having singleton deficiency index one, if and only if *r*_*2*_*, …,r*_*r*_ are all nonnegative and Equation 28 holds for *r*_*1*_.

Suppose now that the singleton deficiency index is 2 or higher. One can employ an inductive approach to constructing computable lower bounds for the negative coefficients in Equation 24. More specifically, assume that computable lower bounds are known for singleton deficiency index *d* and below and consider the problem of computing them for singleton deficiency index *d+1.* The vector inequality to be analyzed is ***0*** *< r*_*1*_***a***_*1*_ *+ … + r*_*d +1)*_ ***a***_*(d +1)*_ *+ r*_*(d+2)*_***BP***_***(****d****+****2****)***_ *+ … + r*_***r***_***BP***_*r****.***_***.*** Appealing to the inductive hypothesis, the only interesting case to consider is when all the coefficients of *w*_*j1*_*,*_*…*_*,w*_*j(d+1)*_ are negative giving rise to the system of inequalities

(Equation 29) $$\left|r_{1}\right|w_{j1}+\ldots +\left|r_{\left(d+1\right)}\right|\;w_{j\left(d+1\right)}<c_{j}≔r_{\left(d+2\right)}w_{j\left(d+2\right)}+\ldots +r_{r}w_{jr}\;for\;j=1,\ldots ,n\text{.}$$

The basic strategy for computing lower bounds on the negative coefficients used above for the case *d=1* applies to the general case Equation 29, the primary difference being that the geometry becomes a bit more complicated in higher dimensions. The key idea is to compute the desired lower bounds on the negative coefficients for each fixed value of *j* in Equation 29 and then minimize over the set of all *j* for which the coefficients *w*_*j1*_*,*_*…*_*, w*_*j(d+1)*_ are all strictly positive. It is helpful to first view the inequalities Equation 29 geometrically and then algebraically. To that end, the geometric problem corresponding to Equation 29 is to find all points (*|r*_*1*_*|,*_*…*_*,|r*_***(****d****+****1****)***_*|*) in the positive orthant of *(d+1)*-dimensional Euclidean space satisfying the inequality Equation 29. It is easily seen that these points lie in the bounded region in the positive orthant of *(d+1)*-dimensional Euclidean space defined by slicing the positive orthant by the hyperplane

(Equation 30) $$\left|r_{1}\right|w_{j1}+\ldots +\left|r_{d+1}\right|w_{j\left(d+1\right)}=c_{j}$$

This defines a (*d+2)-*sided polyhedral set in the positive orthant of *(d+1)*-dimensional Euclidean space bounded by the *d+1* coordinate hyperplanes and the oblique hyperplane define by Equation 29. The geometric solution to the full set of inequalities Equation 29 solved simultaneously over the set of all *j* for which the coefficients *w*_*j1*_*,*_*…*_*, w*_*j(d+1)*_ are all strictly positive is given by intersection of all these (*d+1)-*sided polyhedral sets that will give a bounded set in the positive orthant of *(d+1)*-dimensional Euclidean space with *d+1* “flat” faces lying in the coordinate hyperplanes and a hypersurface cutting through the positive orthant. Generalizing what was done above for the case *d=1,* this hypersurface can be easily calculated by introducing generalized polar/spherical coordinates in *(d+1)*-dimensional Euclidean space. More specifically, consider a parametric representation of the unit *d-*sphere centered at the origin in *(d+1)*-dimensional Euclidean space taking the form

(Equation 31) $$x_{j}=r\;g_{j}\left(q_{1},\ldots ,q_{d}\right),for\;j=1,\ldots ,d+1,satisfying\;g_{1}{\left(q_{1},\ldots ,q_{d}\right)}^{2}+\ldots +g_{\left(d+1\right)}{\left(q_{1},\ldots ,q_{d}\right)}^{2}=1\text{.}$$

It follows from Equation 31 that the radial coordinate *r* satisfies the relationship *r*^*2*^ *= x*_*1*_^*2*^*+*_*…*_ *+x*_*(d+1)*_^*2*^*.* Substitution of Equation 31 into the hyperplane Equation 29 (making the obvious notational change *x*_*j*_ *=* |*r*_j_|) gives after suitable rearrangement

(Equation 32) $$r_{j}\left(q_{1},\ldots ,q_{d}\right)=c_{j}/\left(g_{1}\left(q_{1},\ldots ,q_{d}\right)a_{1j}+\ldots +g_{\left(d+1\right)}\left(q_{1},\ldots ,q_{d}\right)a_{\left(d+1\right)j}\right)$$

as the representation of the hyperplane Equation 29 in the chosen generalized polar/spherical coordinates Equation 30. One now readily sees that the system of inequalities Equation 29 holds provided

(Equation 33) $$r<r_{\mathrm{Min}}\left(q_{1},\ldots ,q_{d}\right)=\min\left\{r_{j}\left(q_{1},\ldots ,q_{d}\right)\right\}$$

where the minimum is taken over the set of all *j* for which the coefficients *w*_*j1*_*,*_*…*_*, w*_*j(d+1)*_ are all strictly positive. The function *r*_*Min*_*(q*_*1*_*,*_*…*_*,q*_*d*_*)* in Equation 33 gives a parametric representation for the desired *d-*dimensional hypersurface. Appealing to Equation 31, it follows that points (*|r*_*1*_*|,*_*…*_*,|r*_***(****d****+****1****)***_*|*) simultaneously satisfy Equation 29 if and only if Equation 33 holds.

We now resume investigating the role that singleton theory plays in representing a general nonnegative pathways ***v*** as a linear combination Equation 17 of the basic pathways ***BP***_*1*_*, …,****BP***_*r*_. In vector/matrix notation the problem is: Find an *r-*dimensional vector ***R*** *=(r*_*i*_*), I=1, …,r* satisfying

(Equation 34) P=WR.

If ***W*** is singleton complete, the answer is given by Equation 20

(Equation 35) $$r_{j}=p_{i\left(j\right)}/w_{i\left(j\right)j}$$

Since each *r*_*j*_ in Equation 19 is nonnegative, Equation 17 gives each nonnegative pathway as a weighted sum of the basic pathways ***BP***_*1*_*, …,****BP***_*r*_.

Suppose now that ***W*** is singleton deficient with deficiency index 1. If necessary, rearrange the columns of ***W*** so that the first column is the one with no singleton entry. Hence, every column vector ***BP***_*j*_ for *j=2, …,r* has at least one singleton entry and every non-zero entry of the first column vector ***BP***_*1*_ is in a row with at least two or more non-zero entries. The interpretation of the representation Equation 17 reduces to the two cases *r*_*1*_ *> 0* and *r*_*1*_ *< 0.* For *r*_*1*_ *> 0,* the representation Equation 17 expresses the steady-state pathway ***v*** as a weighted sum of the basic pathway column vectors ***BP***_*j*_ for *j=1, …,r* as in the singleton complete case*.* For the case *r*_*1*_ *< 0,* one can view Equation 17 in the form

(Equation 36) $$v=\left|r_{1}\right|{BP}_{1}+v_{1}$$

with

(Equation 37) $$v_{1}=r_{2}{BP}_{2}+\ldots +r_{r}{BP}_{r}$$

The pathway vector ***v***_*1*_ is a weighted sum of the basic pathway column vectors ***BP***_*j*_ for *j=2, …,r,* and as discussed previously, the weight |*r*_*1*_| must be restricted in magnitude so that ***v*** is a nonnegative pathway vector. It follows that one can interpret Equation 35 as expressing ***v*** as the weighted sum of ***v***_*1*_ in Equation 37 *diminished* by the weighted basic pathway **|*r***_*1*_|***BP***_*1*_. Indeed, every non-zero entry in ***BP***_*1*_ has a corresponding non-zero entry in ***v***_*1*_ and that *r*_*1*_ must chosen so that ***v*** in Equation 36 has only non-negative entries. Thus, ***v*** in Equation 36 arises by reducing (possibly to zero) the magnitudes of some of the positive entries of ***BP***_*1*_ through -|*r*_*1*_| ***BP***_*1*_.

Solving the system Equations 36 and 37 for the rates *r*_*2*_*, …,r*_*r*_ proceeds exactly as in the singleton complete case discussed above since all of the column vectors ***BP***_*j*_ for *j=2, …,r* have at least one singleton entry. Solving for the remaining weight *r*_*1*_ is readily accomplished by using any of the equations in the system Equation 36 in which *r*_*1*_ appears. For example, suppose that the basic pathway vector ***BP***_*1*_ has the non-zero entry *w*_*i1*_. Then the *i*^*th*^ equation of the system Equation 36 takes the form *v*_*i*_ *= r*_*1*_ *w*_*i1*_ *+ r*_*2*_ *w*_*i2*_ + … + *r*_*r*_ *w*_*ir*_ which can be solved for *r*_*1*_ giving *r*_*1*_ *=*(*v*_*i*_ *– r*_*2*_ *w*_*i2*_ - … - *r*_*r*_ *w*_*ir*_)/*b*_*i1*_ with the rates *r*_*2*_*, …,r*_*r*_ already specified as functions of *v*_*i*_*, i=1, …,n.* With all of the rates *r*_*1*_*, …,r*_*r*_ now known as functions of *v*_*1*_*, …,v*_*n*_, substitution back into Equation 34 gives the required *m* compatibility relations among *v*_*1*_*, …,v*_*n*_.

The extension of these arguments to the general case of singleton deficiency index *d > 0* for ***W*** is straightforward. Rearranging the columns of ***W*** if necessary so that its first *d* columns are the only columns lacking a singleton entry, the most interesting case is

(Equation 38) $$p=-\left|r_{1}\right|{BP}_{1}-\ldots -\left|r_{d}\right|{BP}_{d}+v_{d}$$

with

(Equation 39) $$v_{d}=r_{d+1}{BP}_{d+1}+\ldots +r_{r}{BP}_{r}$$

The representation Equation 38 has the interpretation of the pathway vector ***v*** being expressed as the weighted sum, ***v***_*d*_*,* of basic pathways diminished by the weighted sum of basic pathways **|*r***_*1*_| ***BP***_*1*_ + … ***+* |*r***_*d*_| ***BP***_*d*_. Because the basic pathway vectors ***BP***_*j*_ for *j=d+1, …,r* all possess at least one singleton entry, the rates *r*_*d+1*_*, …,r*_*r*_ are readily calculated as above and the remaining rates *r*_*1*_*, …,r*_*d*_ are easily found by solving the reduced linear system Equation 38. In practice, the deficiency index is usually a small number making the linear system that must be solved for *r*_*1*_*, …,r*_*d*_ of small dimension.

### Case study

As a case study, a simple symbolic model was formulated that couples *glycolysis* and the *tricarboxylic acid (TCA)* or *Citric Acid* cycle*.* This model, similar to one developed by Bordbar et al. 2014,^3^ is used to illustrate how to construct and describe basic pathways, and their use in understanding the workings of a metabolic network in a growing cell. Both the model below and the one in Bordbar et al. 2014^3^ include glycolysis linked to the TCA cycle. The importance of these two pathways is evident from the fact that most daily food calories originate from the catabolism of carbohydrates, which employs glycolysis. Respiration, the process that generates most of the ATP used for human energy, utilizes NADH as produced mainly by the TCA cycle.

#### Case model formulation

Glycolysis occurs in the cytosol whereas the TCA cycle occurs in mitochondria. Glucose, a 6-carbon sugar obtained from the hydrolysis of carbohydrates, is converted, via glycolysis, into two molecules of pyruvate, a 3-carbon metabolite. During this process, there is a *net* gain of 2 ATP’s per glucose (via substrate-level phosphorylation of ADP and phosphate ions). We emphasize *net* because there is an initial energy investment, namely the hydrolysis of 2 ATP’s per glucose, used to activate glucose for subsequent thermodynamically-spontaneous catabolic reactions. Indeed, glycolysis is typically divided into an “investment” phase and a “payback” phase. The latter commences after a 6-carbon metabolite is split into two 3-carbon fragments. Payback also involves the oxidation of carbon, generating two NADHs from the two-electron reduction of two NAD^+^ molecules.

The fate of pyruvate depends on the availability of O_2_. During low O_2_ (anaerobic) conditions, as occurs while vigorously exercising, pyruvate becomes reduced to lactate, generating more NAD^+^. This allows additional glucose to be catabolized and ATP to be generated. If not for this reaction, the rate of glycolysis (and substrate-level ATP generation) would decline due to insufficient NAD^+^.

When O_2_ is plentiful, pyruvate transfers into mitochondria, where it becomes oxidatively decarboxylated and thioesterified, forming CO_2_ and the two-carbon metabolite acetyl-coenzyme A. Acetyl-coenzyme A flows into the TCA cycle. During each turn of the cycle, the acetyl portion of acetyl-CoA becomes attached to a preexisting 4-carbon metabolite, and the resulting 6-carbon unit is oxidized through a series of steps that generate two additional CO_2_ molecules and regenerate the original 4-carbon metabolite. Reducing equivalents in the form of NADH are generated in the process and they are passed to respiratory complexes in the inner mitochondrial membrane where they ultimately react with O_2_ in a series of thermodynamically favorable steps that indirectly generate ATPs via oxidative phosphorylation. Considered collectively, this is respiration.

**Figure 1:**
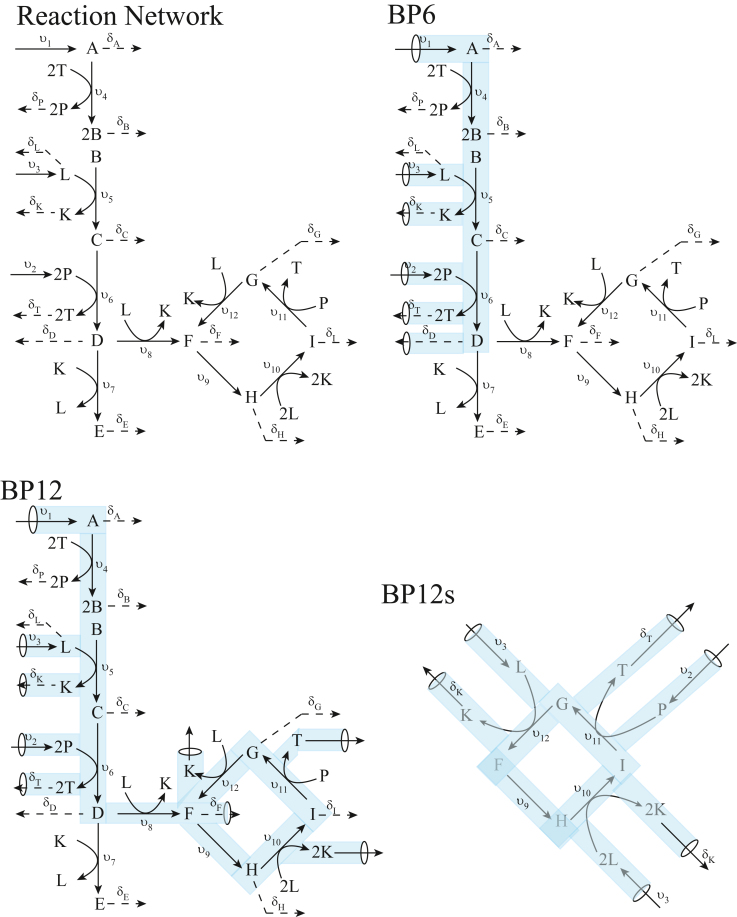
Model of glycolysis and the TCA cycle Three basic pathways are highlighted in blue including BP6, BP12, and BP12s. Rates for non-dilution reactions are designated v_1_, …,v_12_ whereas dilution rates are designated by δ_C_ where the subscripted *C* refers to each component of the model.

The reaction network for the considered model is illustrated in the upper left side of Figure 1. The linear pathway on the left side of the network reflects glycolysis whereas the diamond-like portion on the right represents the TCA cycle. The reaction network is presumed to operate within a cell growing at an exponential rate. Following Bordbar et al. 2014,^3^ model components include A, B, C, D, E, F, G, H, I, K, L, T, and P. Components K, L, T, and P represent NADH, NAD^+^, ATP, and ADP, respectively. Each component must be either imported as a nutrient or generated within the cell from imported nutrients. Counterbalancing this, cell growth promotes the dilution of each component.

The pathway begins with the import of glucose (symbolized A), NAD^+^, and ADP. The investment phase of glycolysis is symbolized by the single reaction {A + 2T → B + 2P} where B represents glyceraldehyde-3-P. The payback phase is reflected by two reactions, the first of which {B + L → C + K} oxidizes glyceraldehyde-3P into 1,3-bisphosphoglycerate (symbolized C) as NAD^+^ is reduced to NADH. This is followed by a lumped reaction {C + 2P → D + 2T} in which pyruvate (D) is formed as ATP is generated from ADP.

Pyruvate has two potential fates, depending on the NADH/NAD^+^ ratio. Under aerobic conditions, the ratio is low because another series of reaction, not included here, uses O_2_ to reoxidize NADH to NAD^+^ while condensing ADP and H_2_O to make ATP. Under these conditions, pyruvate tends to flow into the TCA cycle, reflected in the model by reaction {D + L → F + K} where F is citrate. Under anaerobic conditions, the NADH/NAD^+^ ratio is high, and pyruvate tends to become reduced to lactate, modeled here as {D + K → E + L} where E is lactate. In this case, lactate levels rise, generating the burning sensation associated with vigorous exercise.

Under most cases, the TCA cycle operates in the counterclockwise direction. Citrate is converted to isocitrate (symbolized H) and isocitrate is converted to succinyl-CoA (symbolized I). The major product of the next reaction {I + P → G + T} G represents succinate, fumarate, and malate combined. The cycle completes when this combined species converts to citrate via the reaction {G + L → F + K}.

#### Basic pathways analysis

**Figure 2:**
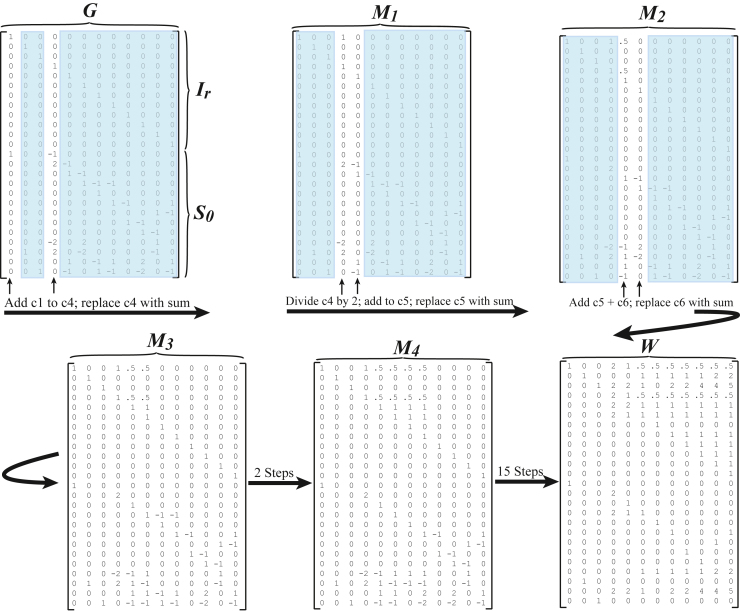
Illustration of how Singleton theory is used to transform matrix ***G***, for the model of Section Case study, into matrix ***W***

**Figure 3:**
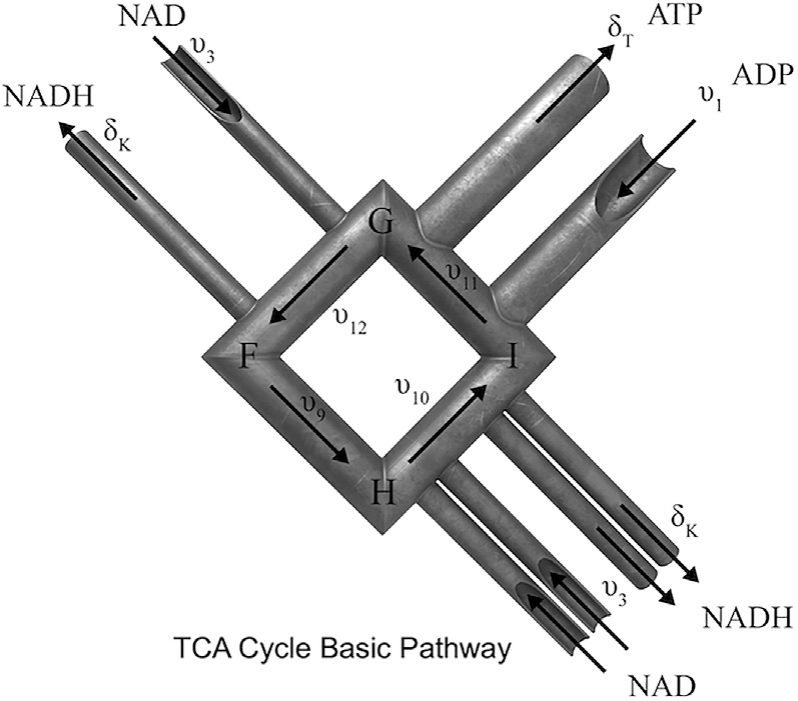
Welded-Pipe illustration of Basic Pathway BP12s The TCA cycle is symbolized by the diamond core. Substrates NAD and ADP enter the cycle, and products NADH and ATP exit, as shown. Each pipe section symbolizes a reaction in the model, with diameters and number of pipes related to the coefficients given by the ***W*** matrix. Molecular species are viewed as flowing through the pipe section under steady-state conditions.

Consider first a version of the model introduced above with all reactions being irreversible. The stoichiometric matrix ***S*** has the form Equation 9 with *m=13* and ***S***_*0*_ the (*13* X *12)-*dimensional matrix in Table S1. A linear basis for the null-space of ***S*** is then given by the columns of the (25 X *12)-*dimensional matrix ***G*** in Equation 14 with *r=12* being the dimension of the null-space of ***S***. The matrix ***G*** is readily seen to have singleton row index *13* and its sparsity, defined to be the proportion of its entries that are zero, is *0.84.* Note also that ***G*** matrices of the form Equation 14 are singleton complete, that is, each of its columns contains at least one singleton row entry. The algorithm for computing the basic pathways matrix ***W*** from ***G*** presented in Section Results gives the matrix in Table S2. The process is illustrated in Figure 2. This matrix ***W*** has singleton row rank 11 implying that the singleton row cost of transforming ***G*** into ***W*** is 2. It also has singleton column deficiency index equal to 2, meaning that all but 2 of its columns have at least one singleton row entry, specifically only columns ***BP6*** and ***BP8*** lack a singleton row entry. Also, a simple calculation gives that the sparsity of ***W*** is 0.84. It is also worth noting, that by applying the simple rank test in (Jevremovic et al. 2008^35^), one can show that all of the 12 basic pathways in ***W***, except for ***BP12***, are also extreme rays of the pointed polyhedral flux cone. It merits noting that ***BP12*** is the only of the twelve basic pathways to contain the four reaction rates *v*_*9*_*, v*_*10*_*, v*_*11*_ and *v*_*12*_ corresponding to the full TCA cycle. Interestingly, if ***BP12*** is replaced by the difference ***BP12-BP8***, then the new ***BP12*** has the simpler form

(Equation 40) $$BP12s={\left(0,1,3,0,0,0,0,0,1,1,1,1,0,0,0,0,0,0,0,0,0,1,0,3,0\right)}^{T}$$

which also includes the full TCA cycle but no glycolysis reactions (Table S2). This basic pathway is illustrated as a series of welded pipes in Figure 3. ***BP12s*** is also readily seen to be an extreme pathway. Thus, the columns of the simplified matrix ***W****s* containing the simplified column vector ***BP12s*** in Equation 40 form a linear basis for the stoichiometric null-space consisting exclusively of extreme pathways for the associated convex polyhedral flux cone. This does not mean that the columns of ***W****s* constitute a frame for the flux cone since there might be many additional extreme rays besides the twelve columns of ***W****s*. That the columns of ***W****s* happen to all be extreme pathways of the flux cone is a serendipitous outcome of the basic pathways algorithm plus the observation leading to Equation 40. Indeed, since the basic pathways algorithm obviously has computational complexity that scales as a polynomial of system size and the problem of finding all extreme pathways of the flux cone is NP-hard, the basic pathways algorithm cannot be expected, in general, to produce a matrix ***W*** all of whose columns are extreme pathways. Finding extreme rays of the flux cone is not a goal of the basic pathways algorithm; rather, it is designed to find a null-basis of the stoichiometric matrix consisting of (nonnegative) pathways in the flux cone.

Suppose now that one wishes to write a generic pathway ***v*** in the pointed polyhedral flux cone corresponding to this metabolic system as a linear combination of the basic pathways (columns of ***W****s*), that is, one wishes to find a solution ***c*** to the algebraic system

(Equation 41) v=Ws.r.

Then making use of the singleton complete columns of ***W****s*, one sees that r_*j*_*, j=1,…12, j* ≠ *6,* take the values

(Equation 42) $$r_{1}=v_{13},r_{2}=v_{23},r_{3}=v_{25},r_{4}=v_{14}/2,r_{5}=v_{15},r_{7}=v_{17},r_{8}=v_{18},r_{9}=v_{19},r_{10}=v_{21},r_{11}=v_{19},r_{12}=v_{12}\text{.}$$

The remaining value r_*6*_ comes from the system Equation 41 giving

(Equation 43) $$r_{6}=v_{16}-v_{14}=v_{15}$$

The system Equation 41 has 25 equations, 12 of which give the values in Equations 42 and 43. The remaining 13 equations in Equation 41 yield the compatibility relations listed in Table S3. Subsequent substitution of the values given in Equations 42 and 43 into the relations in Table S3 produces the 13 compatibility relations among the pathway components *v*_*1*_*, …,v*_*25*_ listed in Table S4. It follows that one can take as *independent parameters* the 12 pathway rates *v*_*12*_*, v*_*13*_*, v*_*14*_*, v*_*15*_*, v*_*16*_*, v*_*17*_*, v*_*18*_*, v*_*19*_*, v*_*20*_*, v*_*21*_*, v*_*23*_ and *v*_*25*_ while the remaining 13 pathway rates *v*_*1*_*, v*_*2*_*, v*_*3*_*, v*_*4*_*, v*_*5*_*, v*_*6*_*, v*_*7*_*, v*_*9*_*, v*_*10*_*, v*_*11*_*, v*_*22*_ and *v*_*24*_ are *dependent parameters* given as functions of the independent pathway rates.

Attention is now turned to consideration of reaction reversibility. In the introduction, two methods for incorporating reaction reversibility into the analysis that maintain a pointed convex flux cone were discussed. One consisted of simply reversing specified reactions in the stoichiometric matrix ***S*** and the other involved incorporating both a reaction and its reverse into ***S***. The first method will be illustrated by applying it to the reactions *v*_*9*_, *v*_*10*_, *v*_*11*_ and *v*_*12*_ comprising the TCA cycle in Figure 1. Specifically, suppose that reaction *v*_*9*_ is reversed (replacing *v*_*9*_ by its negation) in the stoichiometric submatrix ***S***_*0*_ given in Table S1. One readily notices that one effect of this change is to cause the row corresponding to the component H (isocitrate) to have only nonpositive entries, from which it follows that, at steady-state, the concentration of H must vanish, which is viewed here as a degeneracy. To avoid this degeneracy, it is obvious from the ***S***_*0*_ given in Table S1 that if *v*_*9*_ is reversed then v_10_ must also be reversed, but that too leads to a degeneracy unless *v*_*11*_ and *v*_*12*_ are also reversed. Hence, to avoid a degeneracy, *v*_*9*_ cannot be reversed unless the entire TCA cycle is reversed.

The second method will now be illustrated by the same scenario just considered. The first step is to augment the submatrix ***S***_*0*_ given in Table S1 by adding the reverse reaction -*v*_*9*_ as an additional column between columns *v*_*9*_ and *v*_*10*_. The corresponding basic pathways matrix ***W*** has dimension (26×13). A generic pathway ***v*** has 26 components *v*_*1*_*, …,v*_*26*_. From the 13 compatibility relations among the 26 components of ***v***, one concludes that if *v*_*10*_ is set to zero, then the corresponding component r_*10*_ in the relation Equation 41 is also zero and the remaining 12 basic pathways coincide with those found above for the case of reaction v_9_ being in its positive direction. On the other hand, if *v*_*10*_ is set to zero (corresponding to reversing v_9_) then from the compatibility relations, *v*_*21*_ must also vanish. But *v*_*21*_ is the dilution reaction corresponding to component H, from which it follows that reversing just reaction *v*_*9*_ gives the degenerate system in which the concentration of H must vanish at steady-state. The two methods yield the same conclusion.

Consider now the system in Table S1 with the full TCA cycle {*v*_*9*_, *v*_*10*_, *v*_*11*_ and *v*_*12*_} reversed. The corresponding basic pathways matrix ***W*** is given in Table S5. Using the rank test in (Jevremovic et al. 2008) one shows that all of the basic pathways in Table S5 are also extreme rays of the steady-state convex polyhedral flux cone corresponding to the network in Figure 1 with the TCA cycle in reverse except for **BP9**. As noted above for the case with the TCA cycle in its forward rotation, **BP9** is the only basic pathway containing the four reaction rates corresponding to the full TCA cycle.

As a final example, notice that reversing *v*_*12*_, the final reaction composing the TCA cycle with forward rotation in Figure 1, does not produce a degeneracy as does reversing any of the other TCA reactions *v*_*9*_, *v*_*10*_ or *v*_*11*_. If the stoichiometric submatrix ***S***_*0*_ is modified by replacing the column *v*_*12*_ by its negation, then a corresponding ***W*** matrix of basic pathways takes the form given in Table S6. Using the rank test in Jevremovic et al. 2008^15^ one shows that all of the basic pathways in Table S6 are also extreme rays of the steady-state convex polyhedral flux cone corresponding to the network in Figure 1 with *v*_*12*_ reversed. Thus, the columns of ***W*** are extreme rays of the intersection of the stoichiometric null-space with the positive orthant as well as forming a linear basis for the stoichiometric null-space. It is important to remark that this does not imply that the columns of ***W*** form a frame for the null-space or even for its intersection with the positive orthant. Indeed, ***W*** is easily seen to have singleton row rank 12 but singleton deficiency index 2. In particular, while ***W*** has 12 rows with singleton entries, columns 3 and 6 of ***W*** do not contain a singleton row entry.

## Discussion

The basic pathways (BPs) that form the columns of the various ***W*** matrices constructed for the example networks considered in Section Case study not only form a linear basis for the stoichiometric null-space, but they also have interesting chemical and biological interpretations. For example, for the ***W*** matrix given in Table S2, the basic pathways have the following subnetwork descriptions relative to the full network depicted in Figure 1.

### Description of each BP

The BPs listed in Table S2 can be viewed as elementary steady-state flow-modes through the network. The sources of each BP are nutrients entering the cell; the sink is typically dilution due to cell growth. The derived BPs can be divided into four categories. The first group (BP1, BP2, and BP3) involve the simple import and dilution of nutrients glucose (A), ADP (D), and NAD (L). These BPs function merely to import sufficient nutrients to compensate for dilution due to cell growth. The second group (BP4, BP5, and BP6) involve glycolysis alone. BP4 imports glucose and generates pyruvate (and excess B). This pathway requires the import of L (NAD^+^) and its reduction to K (NADH) as well as the excessive production of B (the utilized half continues through the pathway to generate D (pyruvate)). This perfectly balances the utilization and production of ATP/ADP so no source or sink for these cofactors are required.

BP5 is similar but in this BP, all of B is utilized by flowing into the payback phase of glycolysis. It generates too much C so the excess C must be diluted. Again, NAD^+^ is consumed and NADH is produced with perfect utilization/production of ATP/ADP. However, the strategy for doing this is different; here the strategy is for the investment phase of glycolysis to operate at normal speed, but for the rate of the payback phase to be half-normal such that an excess of ATP is not generated.

BP6 also only involves glycolysis but in this case, both investment and payback phases operate at matched rates such that necessary amounts of B and C are made. Thus, NADH and ATP are both generated as NAD^+^ and ADP were consumed. This is the major BP for glycolysis as described in textbooks, and is illustrated in Figure 1 (upper right).

The third group, containing only one member (BP7) combines glycolysis with anaerobic fermentation, generating excess lactate. This combination operates when there is insufficient NAD and an excess of NADH (due to insufficient O_2_ to oxidize the generated NADH in respiration). In this case, cells reduce pyruvate to lactate so as to oxidize NADH to NAD^+^, thereby allowing more ATP to be generated under anaerobic conditions than would otherwise be the case.

The fourth group involves glycolysis along with building the TCA cycle, one additional intermediate for each subsequent BP. Thus, BP8 includes only F; both ATP and NADH are generated – with twice as much NADH as when glycolysis operates alone. BP9 generates both intermediate F and H, BP10 generates F, H, and I (and more NADH), and BP11 generates F, H, I, and G (and more ATP). The TCA cycle is complete in BP12, and for this reason excess F is generated. In this BP, illustrated in Figure 1 (lower left), the maximum amount of NADH and ATP (and excess F) are generated. Finally, the simplified BP12s = BP12-BP8, still contains the complete TCA cycle but without any connection to the glycolysis pathway (Figure 1, lower right; Figure 3). As noted previously, this BP lies along an extreme ray of the pointed, convex polyhedral flux cone, whereas BP12 itself is not an extreme pathway.

As mentioned above, having most or all of the basic pathways constructed for the various versions of the model system in Figure 1 also be extreme rays of the flux cone was not an intended goal of the basic pathways algorithm. Rather the basic pathways algorithm is designed to efficiently find a null-basis of the stoichiometric matrix consisting of (nonnegative) pathways in the flux cone in such a manner that the resulting ***W*** matrix, whose columns form a basic pathways stoichiometric null-basis, has small Singleton Deficiency Index which makes writing a generic pathway as a linear combination of basic pathways a trivial task. For example, the basic pathways matrix ***W*** in Table S2 has singleton row rank 11 and column deficiency index 10 meaning that 11 of its rows have a single non-zero entry and 10 of its columns contain at least one singleton row entry. Since the column entries of ***W*** correspond to system reactions, 10 of the system’s reactions are represented in just one basic pathway. One might well argue that a basic pathway containing a singleton entry is *foundational* to the network in that any generic pathway containing that singleton reaction must contain the entire basic pathway that contains that reaction.

### Sparsity

Sparsity is not an explicit goal of the basic pathways algorithm, yet as observed in the previous section, the sets of basic pathways constructed for each of the example networks studied were all quite sparse null-bases. The reason for this is easy to understand. Firstly, the ***G*** matrix Equation 15 corresponding to any network with stoichiometric matrix of the form 10] is a *fundamental matrix* Coleman and Pothen 1986,^5^ 1987^6^ since it contains an imbedded identity matrix of maximum dimension. Such matrices are often very sparse. Moreover, ***G*** is singleton complete which means one simply reads off the coordinates of a generic null-pathway with respect to the null-basis formed from the columns of ***G*** without needing to solve a linear system of equations. The basic pathways algorithm then makes use of Singleton theory to transform ***G*** into the basic pathways matrix ***W*** keeping its deficiency index (the number of columns without a singleton row entry) small which as a byproduct keeps the sparsity high. In general, ***W*** will not have maximum sparsity since finding a null-basis of maximum sparsity is an NP-hard task (Coleman and Pothen 1986^5^) whereas the basic pathways algorithm scales as a polynomial of the system size.

### Generalization

The basic pathways algorithm makes use of the form Equation 9 for the stoichiometric matrix corresponding to a metabolic network occurring in a cell that experiences steady exponential growth with the matrix -***I***_*m*_ accounting for dilution of the networks components resulting from cell growth. It is easy to see that the basic pathways algorithm readily generalizes to stoichiometric matrices having the form ***S*** = (***S***_*0*_ *| -* $\Lambda_{m}$) where $\Lambda =\text{diag}\left(\lambda_{1},...,\lambda_{m}\right)$ denotes a diagonal matrix with strictly positive entries on the main diagonal. Indeed, the corresponding stoichiometric null-basis matrix ***G*** given in Equation 14 takes the form ***G*** = $\left(\begin{array}{c} I_{r}\\ \cdots \\ \Lambda^{-1}S_{0} \end{array}\right)$ with $\Lambda^{-1}=\text{diag}\left(\lambda_{1}^{-1},...,\lambda_{m}^{-1}\right)$. The next steps in the basic pathways algorithm can now be applied to this more general null-basis matrix ***G*** to derive the associated basic pathways matrix ***W***. Such generalizations might arise in cases in which the various metabolic component species are subject to exponential chemical degradation with or without dilution due to cell growth.

### Non-uniqueness

For a given stoichiometric matrix of the form Equation 9, sets of basic pathways are not unique. One convenient strategy for generating many different sets of basic pathways from the same null-basis matrix ***G*** in Equation 15 is to first permute the rows of the ***S***_*0*_, and then employ the basic pathways algorithm to derive the associated ***W*** matrix followed by applying the reverse permutation to the bottom *m* rows of ***W***. Resulting nonunique sets of basic pathways arise from, and indeed reflect, the inherent complexity of steady-state, metabolic network dynamics, and they collectively provide complementary insights into a metabolic network’s fundamental functional structure.

### Conclusions

This contribution treats steady-state dynamics of a metabolic network within a cell subjected to steady, exponential growth and/or steady, exponential component degradation. Only linear dynamics are considered with the unknown being a steady-state vector of reaction fluxes lying in the stoichiometric null-space. Reversible reactions and pathways are handled in such a way that the set of all admissible steady-state reaction fluxes comprise a pointed, convex, polyhedral, conical subset of the stoichiometric null-space. A solution of the problem is defined to consist of a linear basis for the stoichiometric null-space consisting of admissible fluxes called Basic Pathways. The algorithm used to construct the set of basic pathways scales as a polynomial of the system size in contrast to the NP-hard algorithms employed in the traditional notions of solution that go by the names extreme pathways, elementary flux modes, MEMos, MinSpan, etc, and that therefore suffer from the curse of dimensionality. Even though the basic pathways do not necessarily constitute a frame for the convex, polyhedral cone of admissible pathways, they do admit a simple algorithm of necessary and sufficient conditions on the set of coefficients in linear combinations of basic pathways yielding admissible flux vectors. The basic pathways approach is applied to a metabolic network with 13 components and 25 reactions consisting of a TCA cycle coupled to a glycolysis pathway illustrating how the method handles reaction and pathway reversibility as well as various possible degeneracies. Moreover, each basic pathway is shown to have easily understood chemical interpretation. In a forthcoming contribution, the basic pathways approach is applied to a much larger network (79 components, 169 reaction, 90 dimensional stoichiometric null-space), that models iron metabolism in growing saccharomyces cerevisiae cells and incorporates their compartmented structure, illustrating the polynomial scaling of the computational complexity of the method.

Summarized informally, this study provides a mathematically rigorous way of more deeply understanding metabolic reaction networks operating at steady state within growing cells. It does this by introducing *basic pathways (BPs)*, groups of reactions within a network that operate together “in perfect harmony” under steady state conditions. Each BP describes a multi-reaction pathway in which one group of components are transformed into another. This transformation can be viewed as a *flow* and the corresponding BP as a series of welded pipe sections through which the transformative reaction flows, with each section representing an individual reaction (illustrated in Figure 3. The components of the BP matrix ***W*** dictate which reactions are included in each BP (nonzero entries) and which are not (zero entries). For those that are included, the coefficients further specify the *diameter* of each pipe section (with coefficients > or <1 have proportionately greater or lesser cross-sectional areas). This arrangement ensures that the steady-state flow rate through each pipe section is precisely matched. BPs solve a long-standing problem in the field of Systems Biology, one that has been, perhaps, underemphasized. Reaction networks are generally described by a set of differential equations, each of which describes how the concentration of a component changes in time and what factors control such changes. To achieve the steady-state condition occurring within a growing cell, these equations are set to zero, similar to what is done in deriving the Michaelis-Menten equation. Doing so yields a set of algebraic equations involving the rates of various reactions that ensure steady state with each solution (vector) being called a *pathway* and each nonnegative solution being called an *admissible pathway.* A long-standing challenge in analyzing steady-state reaction network dynamics is to find an *easily computable* set of admissible pathways which generate *all* admissible pathways as *easily computable* linear combinations of the generating pathways. The basic pathways introduced in this contribution fulfill that goal.

The generation of BPs for a given reaction network, using Singleton theory, represents a new “tool” to investigate and understand/analyze metabolic reaction networks on a deeper level. The typical approach to understand complex reaction networks is to decompose/organize them into groups, highlighting those that are considered more important and deemphasizing those of peripheral importance. The decisions involved in this process have been based on thoughtful valid reasoning in light of myriad scientific investigations and results. BPs can be used in the future as a tool for assisting in this process - by revealing additional mathematically based and often unobvious relationships and constraints regarding which reactions function as a group. Thus, the BP concept should prove to be a major advance in the systems biology of cellular metabolic networks.

### Limitations of the study

This study addresses reaction networks that occur within growing cells, and this imposes the requirement that each dynamical (ODE-based) equation describing the time-dependent concentration change of a chemical component includes a term that accounts for the steady exponential decline of concentration due to dilution. Although this might be considered a limitation, the theory can be applied more generally to non-growing cells provided the concentration of each chemical component degrades exponentially in time. Another possible limitation of this study concerns the effort required to interpret each basic pathway of a metabolic network from a biochemical perspective. This issue was not investigated, but the observed high sparsity of the basic pathways matrix suggests that the effort required to interpret basic pathways biochemically does not suffer from the curse of dimensionality. Of course, the accuracy and detail associated with such interpretations will be directly related to the accuracy and detail of the assumed reactions.

## STAR★Methods

### Key resources table

**Table:** 

REAGENT or RESOURCE	SOURCE	IDENTIFIER
**Software and algorithms**		

include original source data for computational studies that are essential to reproduce results	Wolfram Research, Inc.	Mathematica 13.3
Literature cited within the key resources table must be included in the references list.		

### Resource availability

#### Lead contact

Further information and requests for resources and reagents should be directed to and will be fulfilled by the lead contact, Jay Walton (jwalton@tamu.edu), Department of Mathematics, Texas A&M University, College Station Texas 77843-3368.

#### Materials availability

This study did not generate new unique reagents.

#### Data and code availability

• Data: This paper analyzes existing, publicly available data. The application model was informed by the metabolism chapters 17 and 21 of *Biochemistry* by D. Voet and J.D. Voet, 4^th^ edition. J. Wiley & Sons, Hoboken NJ.
• Code: All code generated in this study have been included in the supplemental information. DOIs are listed in the key resources table. Any additional information required to reanalyze the data reported in this paper is available from the lead contact upon request.

#### Algorithmic methods

The methods used in this study are algorithmic. The non-standard algorithms developed in this project are briefly summarized as follows.

• The primary algorithmic assumption is that the network stoichiometric matrix must correspond to a cell undergrowing steady exponential growth or correspond to a metabolic network whose component species degrade at an exponential rate. Components need not have the same exponential degradation rate.
• Only steady-state dynamics are considered and physically feasible pathway vectors contain only nonnegative entries.
• The main algorithms utilize the Singleton Theory of Matrices introduced in the paper.
• The first stage of the main algorithm produces a stoichiometric null basis matrix ***G*** in closed form, that is, from a closed formula that does not require solving a large linear system whose size depends upon the network size. The columns of ***G*** comprise a basis for this stoichiometric matrix null space. This null basis does not, in general, consist of physically feasible vectors since some basis vectors can have negative entries.
• The second stage of the main algorithm uses Gauss elimination inspired methods along with Singleton Theory to transform ***G*** into the null basis matrix ***W*** whose columns, called Basic Pathways, are all physically feasible.
• This algorithm has polynomial computational complexity.
