# Supplementary material for: Basic pathway decomposition of biochemical reaction networks within growing cells
Source: iScience. 2023 Nov 22;27(1):108506. doi: 10.1016/j.isci.2023.108506 (PMC10757263; doi:10.1016/j.isci.2023.108506)
Supplement: Document S1. Tables S1–S6 [file mmc1.pdf]

iScience, Volume *27*

Supplemental information

Basic pathway decomposition of biochemical reaction networks within growing cells

Jay R. Walton and Paul A. Lindahl

Supplemental Information for Walton and Lindahl, 2023

Table of Contents

Table S1. The stoichiometric submatrix S_0 for the model of Section 2.6

Table S2. The W matrix corresponding to the stoichiometric matrix of Table S1.

Table S3. Compatibility relations prior to substitution

Table S4 Compatibility relations after substitution

Table S5 The ***W*** matrix corresponding to the stoichiometric matrix in Table S1 with the TCA cycle (v_9, v_{10}, v_{11}, and v_{12}) reversed

Table S6. The ***W*** matrix corresponding to the stoichiometric matrix in Table S1 with only v_{12} reversed.

Data S1/Methods S1. JRW-PAL-Basic Pathways Code. This Mathematica Notebook implements the Basic Pathways algorithm in the paper as referenced in the Data and Code availability portion of the STAR Methods section.

Table S1. Stoichiometric Submatrix $\boldsymbol{S}_0$ for the model of Section 2.6

	υ_1	υ_2	υ_3	υ_4	υ_5	υ_6	υ_7	υ_8	υ_9	υ_{10}	υ_{11}	υ_{12}
A	1	0	0	-1	0	0	0	0	0	0	0	0
B	0	0	0	2	-1	0	0	0	0	0	0	0
C	0	0	0	0	1	-1	0	0	0	0	0	0
D	0	0	0	0	0	1	-1	-1	0	0	0	0
E	0	0	0	0	0	0	1	0	0	0	0	0
F	0	0	0	0	0	0	0	1	-1	0	0	1
G	0	0	0	0	0	0	0	0	0	0	1	-1
H	0	0	0	0	0	0	0	0	1	-1	0	0
I	0	0	0	0	0	0	0	0	0	1	-1	0
T	0	0	0	-2	0	2	0	0	0	0	1	0
P	0	1	0	2	0	-2	0	0	0	0	-1	0
K	0	0	0	0	1	0	-1	1	0	2	0	1
L	0	0	1	0	-1	0	1	-1	0	-2	0	-1

Table S2. The Basic Pathways matrix ***W*** corresponding to the stoichiometric matrix in Table S1.

BP1	BP2	BP3	BP4	BP5	BP6	BP7	BP8	BP9	BP10	BP11	BP12	BP12s	
1	0	0	2	1	0.5	0.5	0.5	0.5	0.5	0.5	0.5	0	υ_1
0	1	0	0	0	1	1	1	1	1	2	2	1	υ_2
0	0	1	2	2	1	0	2	2	4	4	5	3	υ_3
0	0	0	2	1	0.5	0.5	0.5	0.5	0.5	0.5	0.5	0	υ_4
0	0	0	2	2	1	1	1	1	1	1	1	0	υ_5
0	0	0	2	1	1	1	1	1	1	1	1	0	υ_6
0	0	0	0	0	0	1	0	0	0	0	0	0	υ_7
0	0	0	0	0	0	0	1	1	1	1	1	0	υ_8
0	0	0	0	0	0	0	0	1	1	1	1	1	υ_9
0	0	0	0	0	0	0	0	0	1	1	1	1	υ_{10}
0	0	0	0	0	0	0	0	0	0	1	1	1	υ_{11}
0	0	0	0	0	0	0	0	0	0	0	1	1	υ_{12}
1	0	0	0	0	0	0	0	0	0	0	0	0	δ_A
0	0	0	2	0	0	0	0	0	0	0	0	0	δ_B
0	0	0	0	1	0	0	0	0	0	0	0	0	δ_C
0	0	0	2	1	1	0	0	0	0	0	0	0	δ_D
0	0	0	0	0	0	1	0	0	0	0	0	0	δ_E
0	0	0	0	0	0	0	1	0	0	0	1	0	δ_F
0	0	0	0	0	0	0	0	0	0	1	0	0	δ_G
0	0	0	0	0	0	0	0	1	0	0	0	0	δ_H
0	0	0	0	0	0	0	0	0	1	0	0	0	δ_I
0	0	0	0	0	1	1	1	1	1	2	2	1	δ_T
0	1	0	0	0	0	0	0	0	0	0	0	0	δ_P
0	0	0	2	2	1	0	2	2	4	4	5	3	δ_K
0	0	1	0	0	0	0	0	0	0	0	0	0	δ_L

Table S3. Compatibility relations prior to substitution.

$v_1 = r_1 + 0.5\ r_{10} + 0.5\ r_{11} + 2\ r_4 + r_5 + 0.5\ r_6 + 0.5\ r_7 + 0.5\ r_8 + 0.5\ r_9$
$v_2 = r_{10} + 2\ r_{11} + r_{12} + r_2 + r_6 + r_7 + r_8 + r_9$
$v_3 = 4\ r_{10} + 4\ r_{11} + 3\ r_{12} + r_3 + 2\ r_4 + 2\ r_5 + r_6 + 2\ r_8 + 2\ r_9$
$v_4 = 0.5\ r_{10} + 0.5\ r_{11} + 2\ r_4 + r_5 + 0.5\ r_6 + 0.5\ r_7 + 0.5\ r_8 + 0.5\ r_9$
$v_5 = r_{10} + r_{11} + 2\ r_4 + 2\ r_5 + r_6 + r_7 + r_8 + r_9$
$v_6 = r_{10} + r_{11} + 2\ r_4 + r_5 + r_6 + r_7 + r_8 + r_9$
$v_7 = r_7$
$v_8 = r_{10} + r_{11} + r_8 + r_9$
$v_9 = r_{10} + r_{11} + r_{12} + r_9$
$v_{10} = r_{10} + r_{11} + r_{12}$
$v_{11} = r_{11} + r_{12}$
$v_{22} = r_{10} + 2\ r_{11} + r_{12} + r_6 + r_7 + r_8 + r_9$
$v_{24} = 4\ r_{10} + 4\ r_{11} + 3\ r_{12} + 2\ r_4 + 2\ r_5 + r_6 + 2\ r_8 + 2\ r_9$

Table S3. Compatibility relations.

Table S4 Compatibility relations after substitution.

$v_1 = v_{13} + 0.5\ v_{14} + 0.5\ v_{15} + 0.5\ v_{16} + 0.5\ v_{17} + 0.5\ v_{18} + 0.5\ v_{19} + 0.5\ v_{20} + 0.5\ v_{21}$
$v_2 = v_{12} - v_{14} - v_{15} + v_{23} + v_{16} + v_{17} + v_{18} + v_{19} + v_{20} + v_{21} + v_{23}$
$v_3 = 3\ v_{12} + v_{15} + v_{16} + 2\ v_{18} + 4\ v_{19} + 2\ v_{20} + 4\ v_{21} + v_{25}$
$v_4 = 0.5\ v_{14} + 0.5\ v_{15} + 0.5\ v_{16} + 0.5\ v_{17} + 0.5\ v_{18} + 0.5\ v_{19} + 0.5\ v_{20} + 0.5\ v_{21}$
$v_5 = v_{15} + v_{16} + v_{17} + v_{18} + v_{19} + v_{20} + v_{21}$
$v_6 = v_{16} + v_{17} + v_{18} + v_{19} + v_{20} + v_{21}$
$v_7 = v_{17}$
$v_8 = v_{18} + v_{19} + v_{20} + v_{21}$
$v_9 = v_{12} + v_{19} + v_{20} + v_{21}$
$v_{10} = v_{12} + v_{19} + v_{21}$
$v_{11} = v_{12} + v_{19}$
$v_{22} = v_{12} - v_{14} - v_{15} + v_{16} + v_{17} + v_{18} + v_{19} + v_{20} + v_{21}$
$v_{24} = 3\ v_{12} + v_{15} + v_{16} + 2\ v_{18} + 4\ v_{19} + 2\ v_{20} + 4\ v_{21}$

Table S4. Compatibility relations.

Table S5. The Basic Pathways matrix ***W*** corresponding to the stoichiometric matrix in Table S1 with the TCA cycle (v_9, v_{10}, v_{11}, and v_{12}) reversed.

BP1	BP2	BP3	BP4	BP5	BP6	BP7	BP8	BP9	BP10	BP11	BP12	
1	0	0	2	1	0.5	0.5	0.5	1.5	1.5	0.5	0.5	υ_1
0	1	0	0	0	1	1	1	0	0	0	1	υ_2
0	0	1	2	2	1	0	2	0	0	1	1	υ_3
0	0	0	2	1	0.5	0.5	0.5	1.5	1.5	0.5	0.5	υ_4
0	0	0	2	2	1	1	1	2	2	1	1	υ_5
0	0	0	2	1	1	1	1	2	2	1	1	υ_6
0	0	0	0	0	0	1	0	0	0	0	0	υ_7
0	0	0	0	0	0	0	1	1	1	1	1	υ_8
0	0	0	0	0	0	0	0	1	0	0	0	υ_9
0	0	0	0	0	0	0	0	1	1	0	0	υ_{10}
0	0	0	0	0	0	0	0	1	1	1	0	υ_{11}
0	0	0	0	0	0	0	0	1	1	1	1	υ_{12}
1	0	0	0	0	0	0	0	0	0	0	0	δ_A
0	0	0	2	0	0	0	0	1	1	0	0	δ_B
0	0	0	0	1	0	0	0	0	0	0	0	δ_C
0	0	0	2	1	1	0	0	1	1	0	0	δ_D
0	0	0	0	0	0	1	0	0	0	0	0	δ_E
0	0	0	0	0	0	0	1	1	0	0	0	δ_F
0	0	0	0	0	0	0	0	0	0	0	1	δ_G
0	0	0	0	0	0	0	0	0	1	0	0	δ_H
0	0	0	0	0	0	0	0	0	0	1	0	δ_I
0	0	0	0	0	1	1	1	0	0	0	1	δ_T
0	1	0	0	0	0	0	0	0	0	0	0	δ_P
0	0	0	2	2	1	0	2	0	0	1	1	δ_K
0	0	1	0	0	0	0	0	0	0	0	0	δ_L

Table S7. The Basic Pathways matrix ***W*** corresponding to the stoichiometric matrix in Table S1 with only v_{12} reversed.

BP1	BP2	BP3	BP4	BP5	BP6	BP7	BP8	BP9	BP10	BP11	BP12	
1	0	0	2	1	0.5	0.5	0.5	0.5	0.5	0.5	0.5	υ_1
0	1	0	0	0	1	1	1	1	1	2	1	υ_2
0	0	1	2	2	1	0	2	2	4	4	1	υ_3
0	0	0	2	1	0.5	0.5	0.5	0.5	0.5	0.5	0.5	υ_4
0	0	0	2	2	1	1	1	1	1	1	1	υ_5
0	0	0	2	1	1	1	1	1	1	1	1	υ_6
0	0	0	0	0	0	1	0	0	0	0	0	υ_7
0	0	0	0	0	0	0	1	1	1	1	1	υ_8
0	0	0	0	0	0	0	0	1	1	1	0	υ_9
0	0	0	0	0	0	0	0	0	1	1	0	υ_{10}
0	0	0	0	0	0	0	0	0	0	1	0	υ_{11}
0	0	0	0	0	0	0	0	0	0	0	1	υ_{12}
1	0	0	0	0	0	0	0	0	0	0	0	δ_A
0	0	0	2	0	0	0	0	0	0	0	0	δ_B
0	0	0	0	1	0	0	0	0	0	0	0	δ_C
0	0	0	2	1	1	0	0	0	0	0	0	δ_D
0	0	0	0	0	0	1	0	0	0	0	0	δ_E
0	0	0	0	0	0	0	1	0	0	0	0	δ_F
0	0	0	0	0	0	0	0	0	0	1	1	δ_G
0	0	0	0	0	0	0	0	1	0	0	0	δ_H
0	0	0	0	0	0	0	0	0	1	0	0	δ_I
0	0	0	0	0	1	1	1	1	1	2	1	δ_T
0	1	0	0	0	0	0	0	0	0	0	0	δ_P
0	0	0	2	2	1	0	2	2	4	4	1	δ_K
0	0	1	0	0	0	0	0	0	0	0	0	δ_L

JRW-PAL-Basic Pathways Code

This Mathematica Notebook implements the Basic Pathways algorithm in the paper. The code takes as input a user defined (m x n)-dimensional stoichiometric matrix S of the form [10] in which the (m x r)-dimensional sub-matrix S0, with n=m+r, corresponds to the non-dilution network reactions. The code then outputs a nonnegative (n x r)-dimensional matrix W whose columns are the basic pathways in line [16]. Comments are italicized, Mathematica commands are not italicized. Line numbers refer to the paper.

Set the directory for input and output files.

```
SetDirectory["directory name and path"]
```

Input the (m x n)-dimensional stoichiometric matrix S in the form [10] as an Excel spreadsheet S.xlsx. EVERY ROW OF S MUST HAVE AT LEAST ONE POSITIVE ENTRY. If this is not the case, see the paper for how to proceed.

```
S = Flatten[Transpose[Import["S.xlsx"]], 1];
```

Input the dimensions of the stoichiometric matrix S in [10].

```
m = value;
r = value;
n = m + r;
```

Extract the (m x r)-dimensional submatrix S0 in from S in [10].

```
S0 = ConstantArray[0, {m, r}];
For[j = 1, j <= r, j++, S0[[All, j]] = S0[[All, j]] + S[[All, j]]];
```

Construct the (n x r)-dimensional stoichiometric null-basis matrix G in line [15].

```
idG = IdentityMatrix[r];

G = ConstantArray[0 {n, r}];

For[j = 1, j <= r, j++, G[[j, All]] = G[[j, All]] + idG[[j, All]]];

For[j = 1, j <= m, j++, G[[j + r, All]] = G[[j + r, All]] + S0[[j, All]]];
```

Compute the (n x r)-dimensional Basic Pathways matrix W.

```
W = G;

For[i = 1, i <= m,  i++, {For[j = 1, j <= r, j++,
                    If[W[[r + i, j]] > 0, {bz[r + i] = j, Break[]}]],
                    For[k = 1, k <= r, k++,  If[W[[r + i, k]] < 0,
                      W[[All, k]] = W[[All, k]] +
                      Abs[W[[r + i, k]]]*W[[All, bz[r + i]]]/W[[r + i, bz[r + i]]]]]}]
```

Export Basic Pathways matrix W.

```
Export["W.xlsx", W]
```
